# Dead Cell Discrimination with Red Emissive Carbon Quantum Dots from the Medicinal and Edible Herb *Echinophora tenuifolia*

**DOI:** 10.1007/s10895-025-04286-y

**Published:** 2025-04-05

**Authors:** Naciye Ozdemir, Gamze Tan, Atakan Tevlek, Gulsin Arslan, Gokhan Zengin, Idris Sargin

**Affiliations:** 1 Department of Biochemistry, Faculty of Science, Selçuk University, Konya, 42075 Turkey; 2 Department of Biology, Faculty of Science and Letters, Aksaray University, Aksaray, 68100 Turkey; 3 Department of Medical Biology, Faculty of Medicine, Atilim University, Ankara, 06830 Turkey; 4 Department of Biology, Faculty of Science, Selçuk University, Konya, 42075 Turkey

**Keywords:** Antioxidant capacity, Bioimaging, Cytotoxicity, Dead cell discrimination, Red emissive carbon quantum dots

## Abstract

Accurately determining the viability of cells is crucial for in vitro cell research. Fluorescence-based live/dead cell staining is a highly desirable method to assess cell viability and survival in in vitro studies. We describe a green synthesis method to create red-emissive CQDs from the medicinal and edible herb *Echinophora tenuifolia* using microwave irradiation. We observed that the biocompatibility and photostability of the CQDs are superior. The antioxidant capacity of the CQDs and the plant extract were also investigated using different chemical methods (DPPH, ABTS, CUPRAC, FRAP, PBD, and MCA). The antioxidant capacity of the CQDs was similar to that of the extract of *E. tenuifolia*. Cytotoxicity studies indicate that while the CQDs are not toxic to L929, they exhibit significant toxicity towards HepG2 cells. The CQDs exhibited a strong negative zeta potential (-44.0 mV), which contributed to their selective interaction with dead cells while being repelled by viable cells with intact membrane potentials. The optimal concentration for effective, non-toxic imaging was determined to be 25 µg/mL, as lower concentrations did not produce detectable fluorescence. Differential staining experiments confirmed that CQDs selectively stained dead cells, with red fluorescence observed under the Texas Red filter. Moreover, CQDs exhibited favorable fluorescence intensity and stability, which may offer advantages for long-term and reliable bioimaging applications. In vitro studies on HepG2 and L929 cell lines revealed that the red-emissive CQDs from *E. tenuifolia* can be potentially used in bioimaging.

**Supplementary Information:**

The online version contains supplementary material available at 10.1007/s10895-025-04286-y.

## Introduction

Bioimaging is a modern technique used in research and clinical settings, essential for diagnosing and treating diseases [1]. Advanced bioimaging techniques include methods like PET, MR imaging, scanning probe microscopy, fluorescence microscopy, and X-ray methods. Fluorescence microscopy is vital for understanding cellular processes and has gained popularity in vitro applications thanks to nanotechnologies. Time-lapse fluorescence microscopy has been used to observe superoxide dynamics in human lung cancer cells [2]. MET receptor-targeted fluorescence imaging detected multifocal papillary thyroid cancer [3]. In vivo, N-doped carbon quantum dot nanoparticles loaded with indocyanine green (ICG) showed greater accumulation in melanoma cancer cells during photodynamic therapy than free ICG [4].

Fluorescence microscopy, a key biomedical imaging method, uses fluorescent dyes to stain tissues, cells, or compartments, with confocal microscopes offering sectional views [5]. Imaging cells is a delicate process needing special attention. Many methods involve extensive preparation, making simultaneous imaging unsuitable. Fluorescence-based imaging is sensitive, selective, and versatile, enabling the detection of species that cannot be directly imaged (e.g., hydronium ions). However, added materials may degrade the local area. Various dyes, like propidium iodide (PI), ethidium bromide, Hoechst dye, DAPI, acridine orange (AO), and FITC, are used in fluorescence imaging. It’s crucial to differentiate between live and dead cells during viability studies to assess their relative percentages [6].

Determining the proportion of nonviable cells in a sample is essential for assessing cell culture progress and the effects of freezing and thawing [7]. Most live-dead analyses rely on expensive cytometry-specific markers [8]. Traditional viability dyes like PI are limited by poor photostability, leading to rising interest in non-toxic, low-cost, naturally synthesised carbon nanomaterials as alternative fluorescent dyes in imaging studies. These materials show great promise in addressing the shortcomings of traditional dyes and are attracting scientific interest [9].

Nanomaterials, especially crystalline quantum dots (QDs), are increasingly significant in bioimaging research [10]. Green-synthesised nanomaterials offer advantages over traditional methods, as their synthesis enables using non-toxic or less toxic solvents, thereby reducing the environmental impact of the synthesis process. They also show remarkable structural traits like high crystallinity, narrow size distribution, and excellent monodispersibility [11, 12]. Notably, CQDs from green synthesis are valued for their impressive physical and chemical properties, being sourced from non-toxic, cost-effective, and renewable materials for easy synthesis [13]. Carbon quantum dots (CQDs) are widely used in fluorescence imaging due to their excellent water solubility, chemical stability, adjustable optical features, and sizes under 20 nm. They are also biocompatible and non-toxic [14].

Synthesising CQDs requires various raw materials and methods. There is growing demand for high-quality CQDs with exceptional fluorescence. Researchers are recycling carbon-rich waste materials like eggshell membranes and fruit peels [15, 16]. CQDs have also been derived from other organic sources such as milk [17], coffee [18], and some plant leaves [19, 20]. Converting bio-based materials into CQDs boosts biomass value and reduces the need for external reagents due to high heteroatom content (N, O, P) [21].

CQDs are versatile due to their small size, allowing them to cross biological barriers like the blood-brain barrier and glomerular filtration. Their adjustable properties make them ideal as nanocapsules and nanocarriers for targeted drug and gene delivery [1]. They also have unique optical features that are valuable for bioimaging, serving as probes to track DNA and RNA in real-time and as fluorescent agents in microscopy [1, 22].

Most synthesised CQDs exhibit high quantum yields in blue and green wavelengths, limiting their bioimaging applications [23, 24]. High-efficiency red light-emitting CQDs are essential, as UV light can harm cells [25, 26]. Recent studies show that adjusting reaction conditions or using aromatic compounds can yield red and near-infrared-emitting CQDs [27–29]. However, achieving these efficient CQDs with red fluorescence remains a challenging and time-consuming task [29].

Techniques to synthesise CQDs include hydrothermal, microwave, and electrochemical methods [30]. Producing CQDs involves reactions like pyrolysis, carbonisation, and oxidation. Green synthesis methods use inexpensive, non-harmful materials, making processes eco-friendly [31]. Microwaves provide effective, contactless heating, enabling CQD production in minutes instead of hours. This method supports green chemistry principles, with carbon sources treated in a microwave reactor, with or without solvents, under various conditions [32].

CQDs can be synthesised from either natural or synthetic materials. CQDs derived from natural sources, particularly plants, are especially notable for in vitro imaging [33]. For example, fluorescent CQDs from banana stems can detect Fe^3+^ in HeLa and MCF-7 cells at a concentration of 100 µM Fe^3+^ [34].

This present study aimed to synthesise fluorescent CQDs from the medicinal and edible herb *Echinophora tenuifolia* subsp. *sibthorpiana.* The genus Echinophora belongs to the Umbelliferae family, within the Apioideae subfamily and Echinophoreae tribe. This herb grows from the Mediterranean to Afghanistan. Turkey has six species, three of which are endemic [35]. *E. tenuifolia* is a fragrant herb consumed in Mediterranean cuisine and traditional medicine. The plant is used as a spice and is edible in Mediterranean dishes. This herb is also a popular folk medicine remedy due to its carminative properties that aid with digestion [36].

The present study presents dead cell discrimination using red emissive CQDs from a medicinal and edible herb (*E. tenuifolia*). It also describes an easy and eco-friendly method for synthesising red-emissive CQDs from the aerial parts of *E. tenuifolia* via microwave irradiation in water. The study found that red emissive CQDs might be used as a fluorophore dye for cell staining.

## Experimental

### Materials and Instrumentation

*Echinophora tenuifolia* L. ssp. *sibthorpiana* (called Tarhana Grass in Turkish) samples were collected from its natural environment (coordinates of the plant samples collection site: 37.155532 N, 29.500608E, Golhisar, Burdur, Turkey). The plant’s aerial parts were dried on paper at 21 °C, ground and stored at 4 °C. The dried plant specimens are preserved in the herbarium for further study and reference. H_2_O_2_ solution (30%) and quinine hemisulphate monohydrate were obtained from Sigma-Aldrich.

The Supporting Information file provides details about the instruments used in the study.

### Synthesis of CQDs from *E. tenuifolia*

Optimal conditions for CQD synthesis were determined by irradiating plant powder in a solvent using a microwave device (CEM Mars 5). The operating conditions tested for CQD synthesis were as follows. All CQD samples dispersed in water were filtered prior to fluorescence emission measurements using a Sartorius Minisart microfilter (0.2 μm).

#### Solvent Type

The experiments were conducted separately in both water and ethanol environments to determine the suitable solvent for the synthesis. The plant sample (100 mg) in water or ethanol (20 mL) was subjected to microwave irradiation at 800 W for 5 min. The fluorescence emission of the samples was analysed using fluorescence spectroscopy. Subsequently, the solvent that demonstrated the highest fluorescence emission was chosen to synthesise CQDs.

#### Plant Powder/Solvent Ratio

The plant samples (0.05, 0.1, 0.3 and 0.5 g) in the solvent were exposed to microwave irradiation at 800 W microwave energy level for 5 min, and the fluorescence emission of the synthesised samples was measured by fluorescence spectroscopy. The plant powder/solvent ratio showing maximum fluorescence emission was recorded.

#### Microwave Energy Level

The plant samples (0.05 g in 20 mL of ethanol) were subjected to microwave irradiation for 5 min at 400 W or 800 W energy levels. Microwave treatment at 800 W yielded better fluorescence emission.

#### Microwave Irradiation time

To determine the microwave exposure time, the plant samples were subjected to microwave irradiation for intervals of 1, 3, 5, and 10 min using the specified solvent type, plant powder/solvent ratio, and microwave energy level.

#### Separation and Purification

The separation and purification of CQDs were carried out under optimal conditions. Initially, a plant sample (50.0 mg) was combined with 20 mL of ethanol and exposed to microwave radiation at 800 W for 1 min. The mixture obtained was then gathered, and the solid residue was subsequently separated using filter paper. The excess ethanol in the filtrate was removed using an evaporator. After centrifugation at 4000 rpm for 15 min, the CQDs were separated from the medium. CQDs were purified by dialysis against water (3.500 MWCO). The dialysis water was changed three times daily, and the extra water was removed by heating it in an oven at 80 °C. Afterwards, the CQDs in water were freeze-dried and stored as a solid.

Four reaction parameters (microwave exposure time, the solvent type, the power of microwave, the amount of the plant) were tested to optimise the synthesis of CQDs from the plant *E. tenuifolia*, and the results are shown in Fig S1.

### Fluorescence Quantum Yield of CQDs

The fluorescence quantum yield of CQDs was calculated using quinine sulphate as a reference. The quantum yield of CQDs was calculated using the following Eq (1). 

1 $$\:{\varPhi\:}_{x}\:=\:{\varPhi\:}_{R}\left.\left(\frac{{Grad}_{x}}{{Grad}_{R}}\right.\right)\left.\left(\frac{{{\eta}^{2}}_{x}}{{{\eta}^{2}}_{R}}\right.\right)$$

where *Φ*_*x*_ represents the fluorescence quantum yield of CQDs, *Grad* indicates the slope of the plot of integrated fluorescence intensity versus absorbance (*R*: quinine sulfate, *X*: CQDs), η denotes the refractive index of the solvent, *R* refers to the reference (quinine sulfate), and *X* designates the sample (CQDs). The details are presented in the Supporting Information file [37].

### Cell Maintenance

This study used two types of cell lines (HepG2 human hepatocellular carcinoma cell line and L929 mouse fibroblast cell line). Both cell types were cultured in Dulbecco’s Modified Eagle’s Medium (Capricorn, Germany) supplemented with 10% foetal bovine serum (Gibco, USA), 1% antibiotic-antimycotic (Gibco, USA), and 1% L-glutamine (Sigma Aldrich, Germany). The cells were kept at 37 °C under a humidified atmosphere with 5% CO_2_, and the culture medium was replaced twice to thrice weekly.

### Cell Viability Assay

To determine the cytotoxic effect of CQDs and cell viability, the MTT assay was used following Mosmann’s method [38]. Initially, cells were seeded into 96-well plates at a density of 5 × 10³ cells/well and allowed to grow for 24 h. Afterwards, the cells were exposed to varying concentrations of CQDs in the culture medium and incubated for 24, 48, and 72 h. The control group, which did not contain any CQDs, was considered 100% alive compared to the other group. At the end of each time point, the medium was carefully removed from the wells and replaced with 150 µL of serum-free culture medium mixed with 5 mg/mL of MTT. The plates were placed back in the culture incubator for 4 h. After the incubation period, the MTT solution was removed, and the formazan crystals formed were dissolved by adding 100 µL of DMSO (Sigma Aldrich, Germany). The spectrophotometer (BioTek, USA) was used to measure the absorbance value of the final solutions at 570 nm. The absorbance of the CQDs-treated cells was compared to the control group, and the viability of the control group was considered 100%. The percentage of the cell viability was calculated using the following formula (Eq. 2):

2 $$\begin{aligned}\text{Cell viability}\,{(\%)}=&{\text(\text{Optical}\,\text{density}\,{of}\,{treated}\,{cells})}\\&/(\text{Optical}\,\text{density}\,\text{of}\,\text{control}\,\text{cells})\times100\end{aligned}$$

### Cell Imaging Studies

To validate the results of the MTT test conducted as part of the research, the impact of the CQDs on the cells in a manner that depends on the concentration was observed using light microscopy after the 24th and 72nd h. In this context, images were obtained from different wells using an inverted microscope at the same magnification (Leica, Germany). The impact of CQDs on cell fluorescent staining was investigated in a manner that varied with concentration. A fluorescent microscope was used for imaging studies (Leica DMi8, Germany).

Furthermore, the investigation was carried out to determine the staining capabilities of CQDs on both live and dead cells. This research involved the use of DAPI (25 µg/mL, Elabscience, USA), a stain specifically designed for cell nuclei, Acridine Orange (AO) (25 µg/mL, Sigma Aldrich, Germany), a commonly employed stain for live cells, and prepared CQDs (25 µg/mL). The cells obtained from the liquid nitrogen tank were quickly moved into a culture medium kept at 37 °C to stain the viable cells. Furthermore, the cells were immersed in a growth medium and kept at a temperature of 4 °C for 1 h to evaluate the staining of non-living cells. 10 µL of each dye solution was used, and an equal amount of cells from suspensions of HepG2 and L929 cells (5 × 10^3^ cells/mL) were combined to produce mixtures of DAPI/AO/QDs. Subsequently, 10 µL of stained cells was exemplified from the mixture mentioned above and examined using a fluorescence microscope (Leica DMi8, Germany). The examination involved using DAPI, FITC, and Texas Red filters alone and in combination.

### Antioxidant Assays

The study conducted various antioxidant assays to evaluate the properties of CQDs and plant ethanol extracts. These assays included the 2,2-diphenyl-1-picrylhydrazyl (DPPH) and 2,2’-azino-bis(3-ethylbenzothiazoline-6-sulfonic acid (ABTS) assays, which tested the ability of antioxidants to neutralise free radicals, as well as the ferric reducing antioxidant power (FRAP) and cupric reducing antioxidant capacity (CUPRAC) assays, which measured the reduction capabilities of the CQDs and extracts. The metal chelating ability (MCA) and phosphomolybdenum (PBD) assays were also conducted. The Trolox standard was used for all assays except for MCA, which was measured in terms of equivalent EDTA per gram of extract. The procedures used in this study were described in our previous publication [39].

### HPLC Analysis of the Extracts

The phytochemical composition of the methanol extracts was determined using RP-HPLC-DAD (Shimadzu Scientific Instruments, Kyoto, Japan). The separation procedure was performed at 30 °C on an Eclipse XDB C-18 reversed-phase column (250 mm, 4.6 mm length, 5 μm particle size, Agilent, Santa Clara, CA, USA) under optimised experimental conditions. All details are given in the Supporting Information file.

### Statistical Analysis

The one-way analysis of variance (ANOVA) is utilised when comparing multiple groups. If the ANOVA produces statistically significant findings, additional post hoc analyses are carried out. Tukey’s test was employed when the homogeneity of variance was confirmed, whereas the Games-Howell test was used when the homogeneity of variance was not met. When the data did not follow a normal distribution, non-parametric tests such as the Kruskal-Wallis H and Mann-Whitney U tests were used to analyse differences in group means. The threshold for statistical significance (p) was set at 0.05. In non-parametric tests, the p-value was adjusted by the Bonferroni correction for pairwise comparisons.

## Results and Discussion

### Synthesis of CQDs

**Fig. 1:**
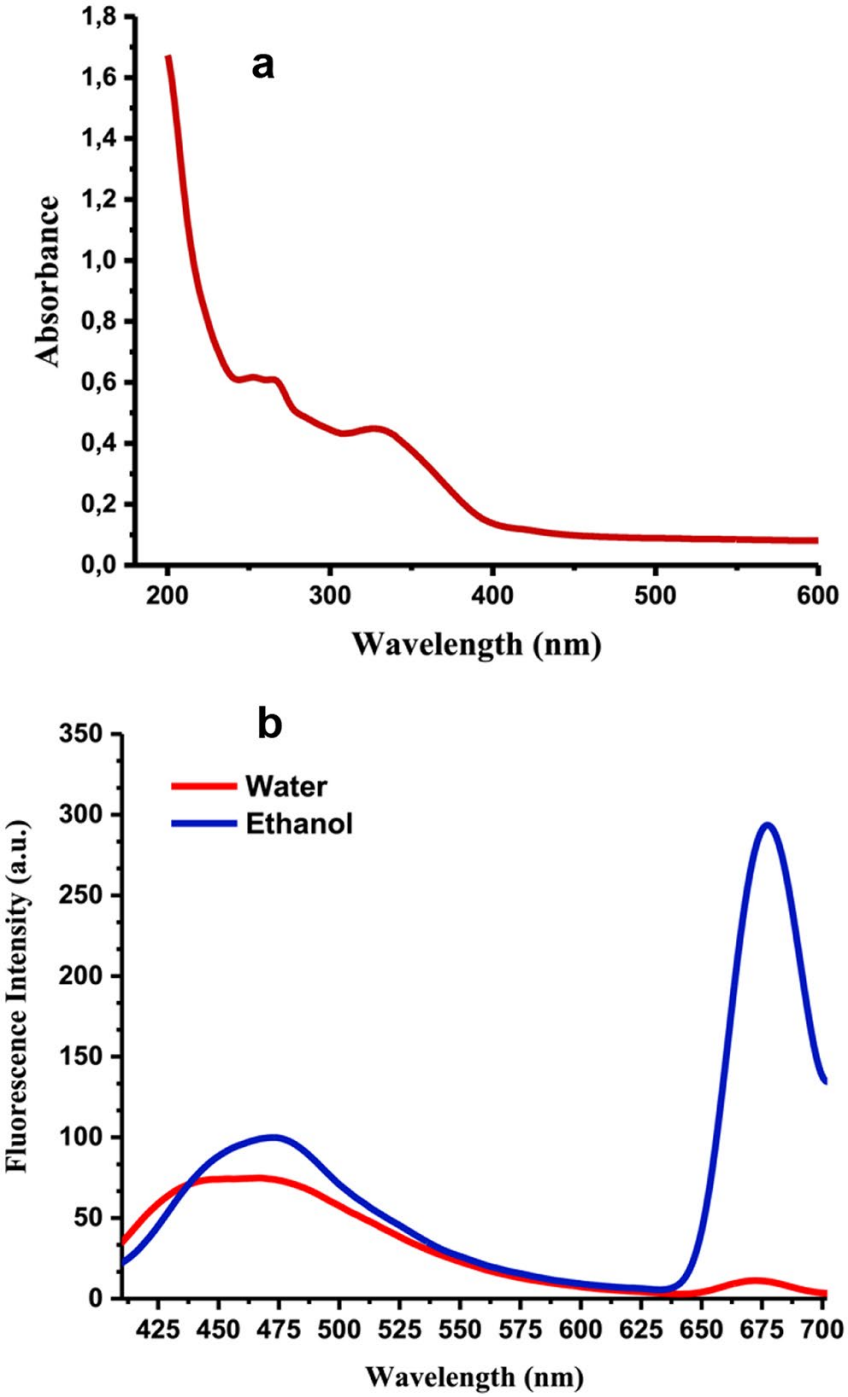
The UV–vis absorption spectrum (**a**) and photoluminescence intensity of CQDs dispersed in water (water: CQDs synthesised in water, ethanol: CQDs synthesised in ethanol) (excitation wavelength 365 nm) (**b**)

Optimum operating conditions for microwave-assisted synthesis CQD from *E. tenuifolia* are defined as follows: solvent type: ethanol, plant powder/solvent ratio: 50 mg/20 mL, energy level: 800 W and irradiation time: 1 min. The light emission efficiency of any substance, known as the quantum yield, is determined by the ratio of emitted photons to absorbed photons. The fluorescence quantum yield for CQDs was measured as 0.027 using quinine sulfate as the reference standard. The absorbance and fluorescence emission spectra of CQDs in an aqueous solution can be seen in Fig. 1a and b.

The electronic band gap transitions of CQDs were investigated using UV-visible spectroscopy. CQDs typically show two absorption peaks, as shown in Fig. 1a. These peaks correspond to the π-π* transition of aromatic sp^2^ domains and the n-π* transition of surface functional groups like carbonyl, hydroxyl, ester, and carboxyl groups [40].

The absorption peaks were detected at 262 and 331 nm, indicating the transitions from π–π* in the sp^2^ hybridised C = C and C = N bonds and the n–π* transition of the C = O bond. It has been previously reported that surface defects are accountable for the broad absorption spectra of CQDs [41]. Due to these surface defects, CQDs absorb UV light within the 200 to 600 nm wavelength range. The graph in Fig. 1b shows the fluorescence intensity emitted by CQDs in an aqueous solution. CQDs solution was excited at 365 nm, and two maxima were recorded at 454 nm and 675 nm wavelengths. Previous studies demonstrated that CQDs that are approximately 2 nm in size exhibit intense blue photoluminescence. As the size of CQDs increases, their fluorescence shifts towards the red end of the spectrum [42]. In other words, depending on their size, the optical characteristics of CQDs can differ [43]. In this study, the solution of the CQDs contains a mixture of small and large sizes (Fig. S2). Small particles may exhibit fluorescence at 454 nm in the blue spectrum, while larger particles could contribute to red fluorescence at 675 nm.

**Fig. 2:**
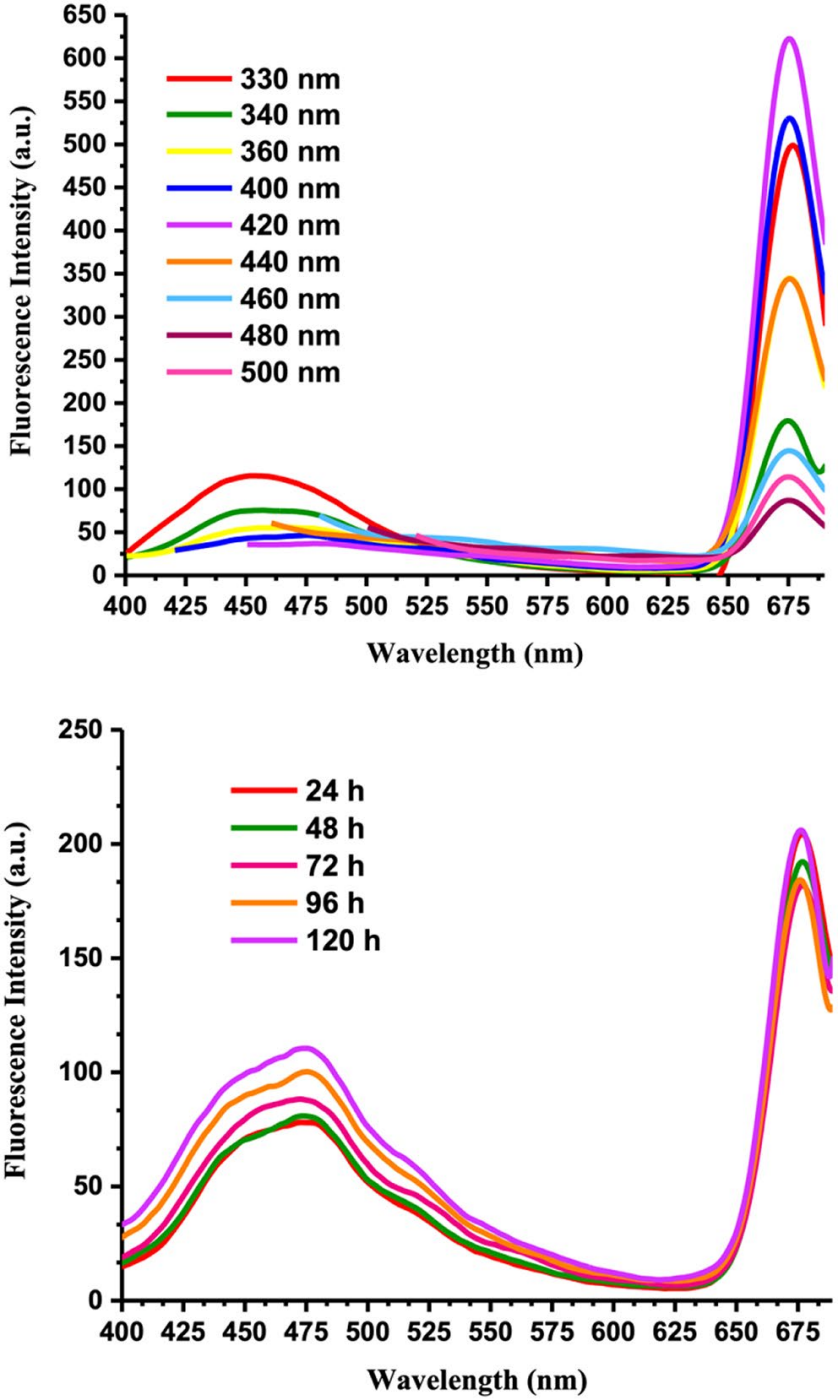
The fluorescence emission intensity of CQDs in an aqueous medium depends on the level of excitation (330–500 nm) (upper spectrum). The fluorescence emission intensity of CQDs in an aqueous solution after 5 days of storage at room temperature (21 °C) (lower spectrum, λ_excitation_: 360 nm)

In the study, it was found that the photoluminescence of CQDs varied with the excitation wavelength in the range of 330 to 500 nm, as depicted in Fig. 2. The CQDs exhibited the greatest fluorescence emission at a 675 nm wavelength when excited at 420 nm. In contrast, the fluorescence emission at 454 nm was higher when the CQDs were excited at 330 nm. Also, it was observed that as the excitation wavelength increased, the peak at 454 nm decreased and moved toward the red end of the spectrum. However, the emission peak at 675 nm did not shift despite the changes in excitation wavelength. A recent study reported similar results for CQDs from banana leaves (Chl-CQDs, Chl: chlorophyll) [44]. The intense peak at 675 nm found in the fluorescence emission spectrum of the Chl-CQDs was associated with chlorophyll molecules located on the surfaces of the CQDs. Surprisingly, this emission peak did not shift toward longer wavelengths despite changes in the excitation wavelength. It is widely accepted that CQDs display excitation-dependent fluorescence emission due to the presence of surface groups, surface defects, and their size [32].

CQDs synthesised in ethanol solution produced red emissions in this study, while those obtained in aqueous solution did not (Fig. 1b). This could be attributed to the chlorophyll molecules from *E. tenuifolia*. Chlorophyll molecules are insoluble in water but can easily dissolve in ethanol. During the synthesis process, CQDs were formed using microwave irradiation at 800 W in a microwave oven, and chlorophylls dispersed in ethanol were fused to CQDs. The CQDs exhibited a prominent fluorescence peak at a wavelength of 675 nm, indicating the presence of chlorophyll molecules on their surfaces. This finding implies that the CQDs can emit red light due to their chlorophyll-rich composition, which aligns with previous research findings [44].

The study analysed how CQDs emit fluorescence with varying excitation wavelengths. CQDs exhibited a weak blue emission peak at 454 nm and a strong red emission peak at 675 nm. The red emission remained constant regardless of the excitation wavelength, indicating the presence of chlorophyll molecules [44, 45]. Furthermore, *E. tenuifolia* contains various types of phenolics, as shown in the HPLC results of the ethanol extract (Table S1). During the synthesis of CQDs, numerous–OH groups were added to the surface of the dots, as confirmed by the stretching band at 3355 cm^–1^ in the FT-IR spectrum (Fig. 4). The presence of a large number of highly electronegative–OH groups in the extract of *E. tenuifolia* contributed to the development of CQDs with red emission [46].

Figure 2 (lower spectrum) presents the results of a photostability test conducted on CQDs in water. The test involved storing the CQDs for 120 h under room conditions (at 21 °C) and observing changes in their photostability over time. The findings showed that the photostability of the CQDs increased over time. This enhanced photostability was likely due to the greater solubility of the CQD particles in aqueous medium.

**Fig. 3:**
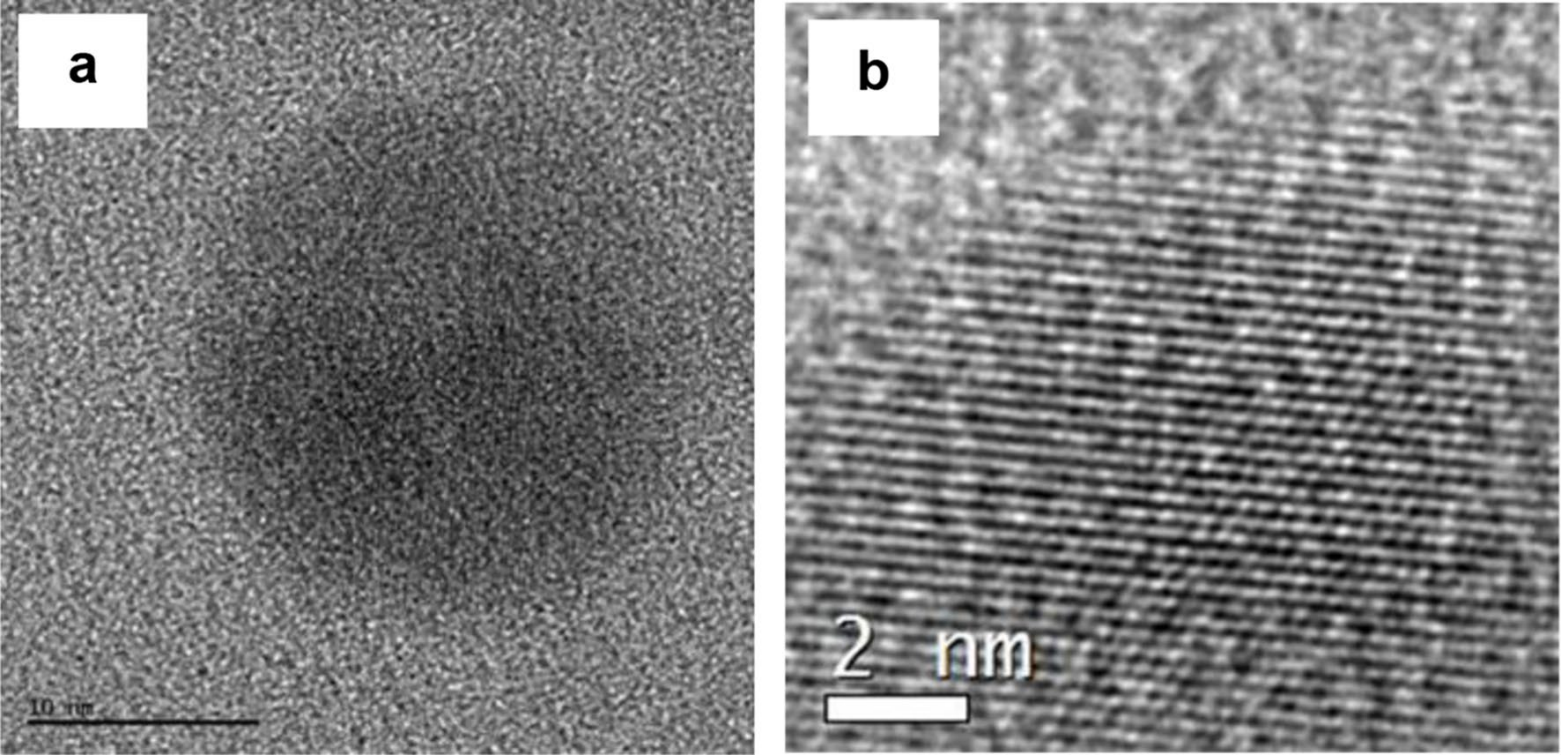
TEM (**a**) and HR-TEM (**b**) images of CQDs obtained from the plant *E. tenuifolia* (Scale bars a: 10 nm, b: 2 nm)

HR-TEM analysis can offer insights into the crystalline or amorphous characteristics and detailed structure of CQDs. The CQDs derived from *E. tenuifolia* are spherical, with a diameter of approximately 20 nm (Fig. S2) (measured using ImageJ software). As depicted in Fig. 3a, the TEM image illustrates the spherical nature of the CQDs. The crystal lattices in the images (Fig. 3b) show that CQDs have a graphitic core with a crystal structure, which is attributed to the graphite planes in the core [47]. The lattice spacing is about 0.26 nm (Fig. S3) (measured using ImajeJ software), which closely resembles that of graphite (~ 0.35 nm), suggesting the crystalline core of the CQDs.

**Fig. 4:**
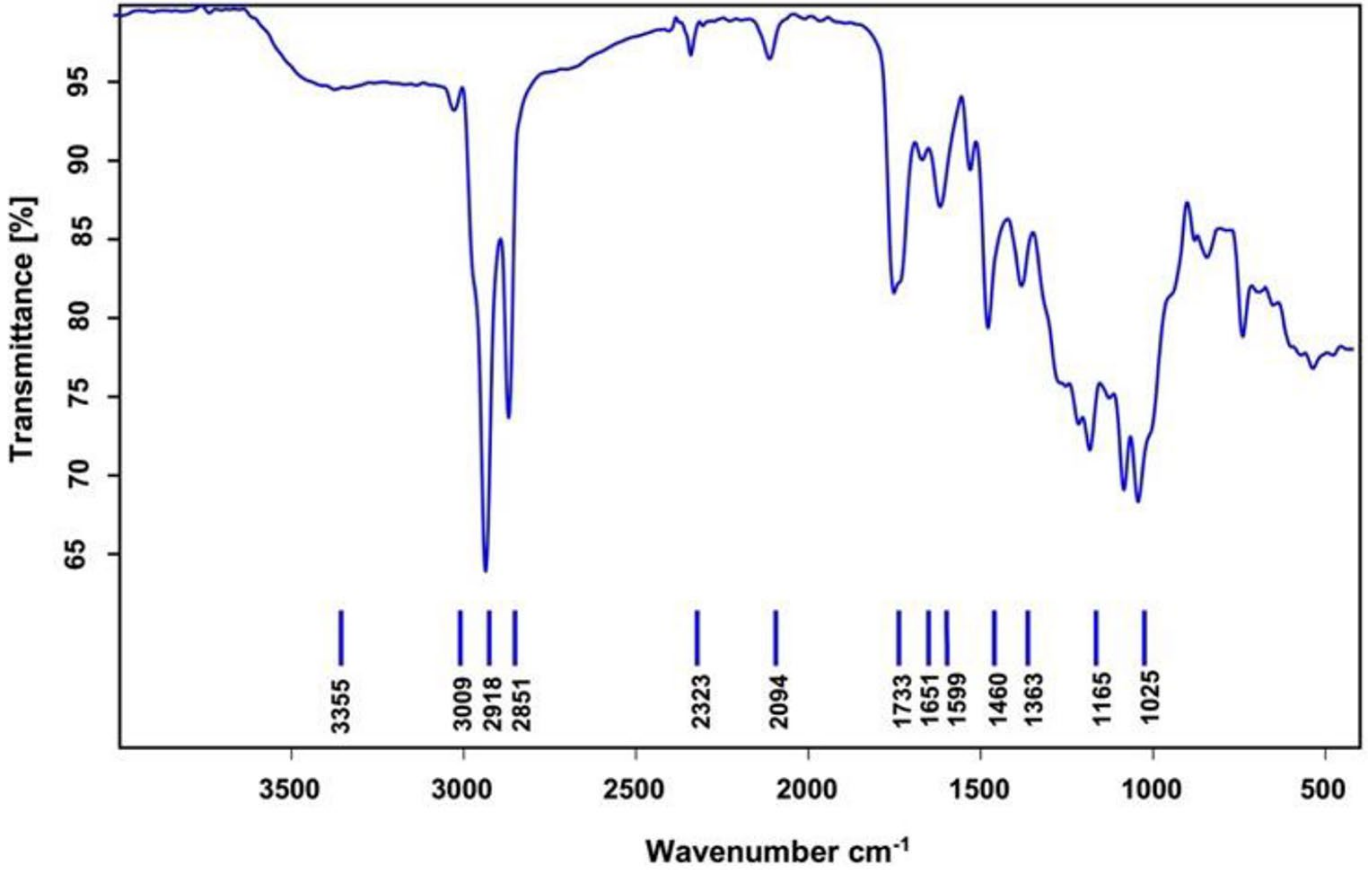
FT-IR spectrum of CQDs derived from the plant *E. tenuifolia*

The FT-IR spectrum of the synthesised CQDs from the plant extract (*E. tenuifolia*) exhibited a stretching vibration band (3355 cm^–1^) due to large–OH groups, confirming the involvement of phenolic molecules with–OH groups in forming carbon quantum dots (Fig. 4). The O-H and N-H stretching vibrations are attributed to the absorption band at 3355 cm^–1^. The 2851 cm^–1^ absorption band corresponds to the–CH stretching vibration. The absorption bands in the 1598–1733 cm^–1^ range are linked to–C = C, C = O, and C = N. Additionally, the absorption band at 1165 cm^–1^ is associated with the–C-O stretching vibration [48].

Also, the CQDs showed a Zeta potential of -44.0 mV, indicating a negative surface charge. The presence of strong electron-withdrawing groups, such as the–OH group on the surface of CQDs from the plant extract (*E. tenuifolia*), made them effective antioxidants and gave them a strongly negative surface charge. This negative charge is one of the reasons why CQDs can distinguish between live and dead cells. Living cells have a high negatively charged membrane potential, which causes them to repel the negatively charged CQDs. This makes it difficult for negatively charged CQDs from *E. tenuifolia* extract to be used in live cell imaging. However, those CQDs can be successfully used for imaging dead cells since membrane potential is only present in living cells [49].

**Fig. 5:**
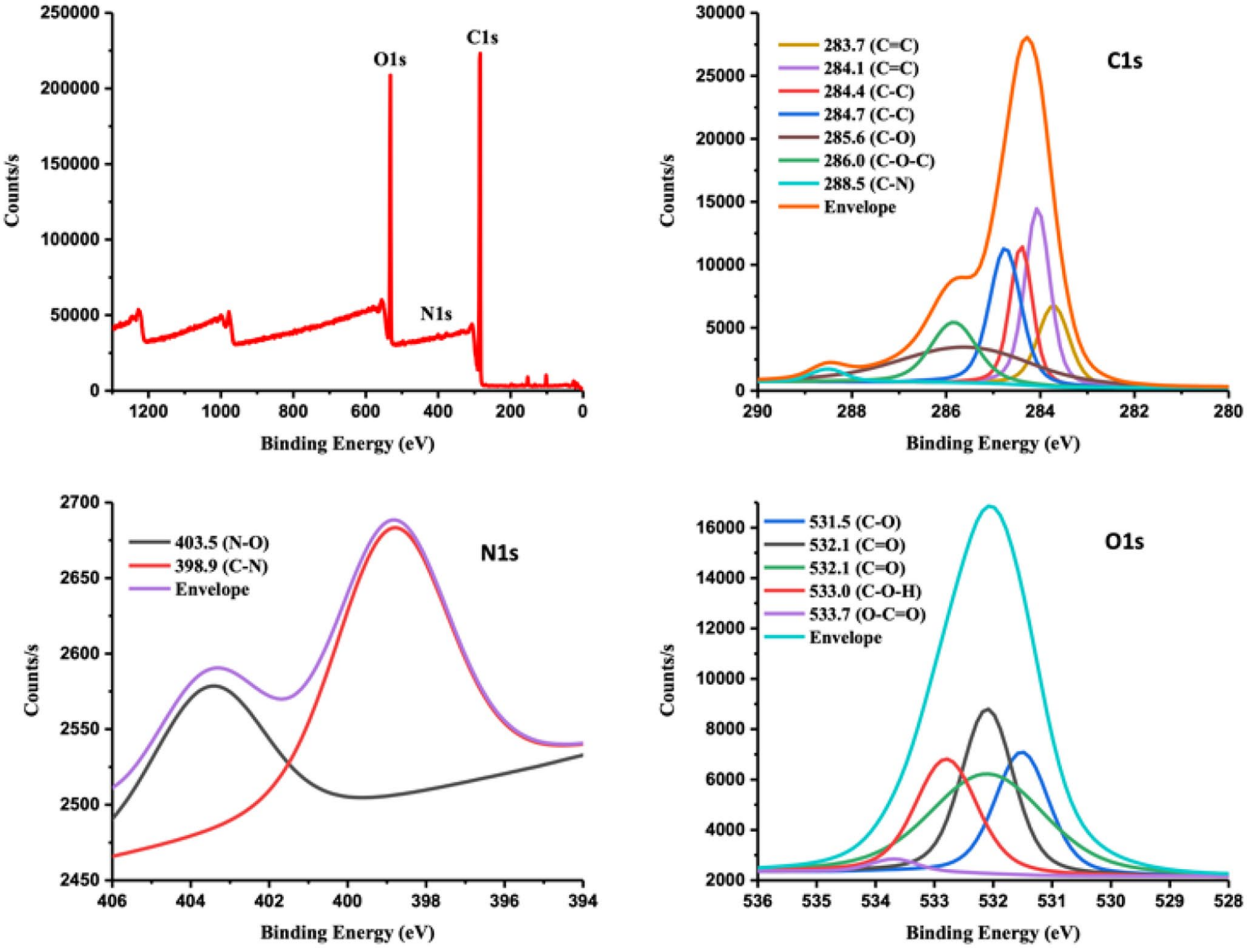
The XPS survey spectrum (top left) of the CQDs obtained from *E. tenuifolia*, along with detailed XPS spectra displaying the signals for C 1s, O 1s, and N 1s. The detailed C 1s, O 1s, and N 1s signals were analysed to identify individual peaks

The structure and chemical bonding of CQDs were examined using XPS analysis. The surface analysis of CQDs revealed the existence of C, O and N, as shown in Fig. 5. The detailed spectra of C1s, N1s, and O1s in Fig. 5 illustrate the identification of carbon bonding linked to the peaks [44, 50]. The peak assignments observed were consistent with the findings of the FTIR analysis, as shown in Fig. 3. The XPS analysis indicated that the CQDs had higher carbon and oxygen content but lower nitrogen content (C: 68.85%, O: 22.96%, N: 0.80%). A comprehensive list of the element percentages obtained from XPS analysis is presented in the Supporting Information file (Table S2).

**Fig. 6:**
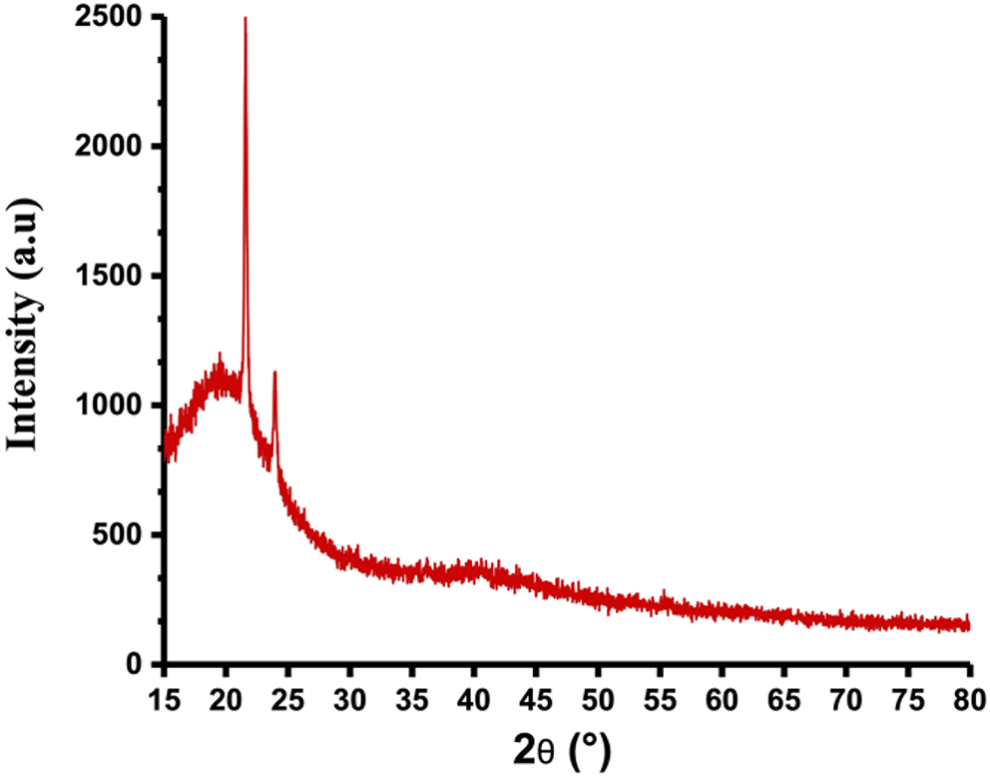
XRD pattern of CQDs derived from the plant *E. tenuifolia*

The XRD analysis is employed to ascertain the chemical composition, particle dimensions, phase purity, and crystal arrangement of CQDs [51]. As illustrated in Fig. 6, the XRD analysis of CQDs revealed a broad peak at 2θ = 20°. This suggests the existence of graphitic carbon crystal planes within the cores of the CQDs. The spacing between the layers suggests that CQDs are less crystalline than graphite because of the existence of functional groups containing oxygen [52]. This suggests an irregularity in the layers between the graphitic cores of CQDs and partial amorphous structure in some parts of the crystal structure. The crystal lattices in the HR-TEM images (Fig. 4b) indicate that CQDs possess a graphitic core with a crystalline structure, attributed to the graphite planes in the core. The CQD diffraction pattern shows peaks at 2θ = 25°, linked to the (0 0 2) planes of carbonaceous materials, indicating disordered carbon atoms in the hexagonal graphite structure, resulting in a turbostratic arrangement through interlayer stacking [53].

### Antioxidant Potential of CQDs

Exploring the antioxidant capacity of both synthesised and natural compounds can provide a better understanding of their potential pharmacological benefits [54]. Various chemical tests were carried out, and the findings are presented in Table 1. The DPPH and ABTS tests are employed to assess the capacity of antioxidants to quench free radicals. If the antioxidants can donate hydrogen to free radicals, they can be classified as chain-breaking antioxidants.

**Table 1:** Antioxidant effects of the CQDs and the tested extract

	CQDs	Extract
DPPH (mg TE/g)	42.62 ± 0.39	44.52 ± 0.60
ABTS (mg TE/g)	46.16 ± 0.82	50.61 ± 3.29
CUPRAC (mg TE/g)	81.06 ± 1.11	85.45 ± 3.87
FRAP (mg TE/g)	45.96 ± 1.24	42.47 ± 1.31
PBD (mmol TE/g)	1.05 ± 0.13	1.22 ± 0.05
MCA (mg EDTAE/g)	17.93 ± 0.41	19.87 ± 0.30

*Values are reported as mean ± SD of three parallel experiments. TE: Trolox equivalent; EDTAE: EDTA equivalent; na: not active

As Table 1 shows, the CQDs (ABTS: 46.16 mg TE/g; DPPH: 42.62 mg TE/g) displayed similar radical scavenging capacity as extract (ABTS: 44.52 mg TE/g; DPPH: 50.61 mg TE/g). The presence of functional groups such as COOH and OH^–^ allows CQDs to scavenge radicals [55–58]. A previous study suggested that compounds characterised by extended conjugated C-C chains typically exhibit strong free radical scavenging properties [59]. Based on the information in Table S1 (showing the phenolic composition of the extract), the primary contributors to its antioxidant capacity were rutin, present at a concentration of 217 mg/g extract, and chlorogenic acid, present at a concentration of 39.6 mg/g extract. Previous studies demonstrated that these compounds have powerful antioxidant properties [60, 61]. Antioxidants’ electron-donating capacity, known as reduction capacity, can be estimated using CUPRAC and FRAP assays. These assays involve converting Cu^2+^ to Cu^+^ and Fe^3+^ to Fe^2+,^ respectively. Like radical antioxidant compounds. The measured values were 1.05 mmol TE/g for CQD and 1.22 mmol TE/g for the extract. Chelating transition metals is important because it helps prevent the creation of hydroxyl radicals in the Fenton reaction, a key aspect of antioxidant mechanisms [54].

The characteristics of CQDs are determined by scavenging assays, the tested CQDs and extract displayed remarkably similar reducing abilities. Notably, the surface groups in CQDs, such as OH, can serve as electron donors, elucidating the observed capability. Like this, phenolics such as rutin and chlorogenic acid, which possess hydroxyl groups, might be responsible for the observed reducing potential [62, 63]. The phosphomolybdenum test involves the reduction of Mo(VI) to Mo(V) at acidic pH due to the precursor substance and the method of synthesis employed in their production [14]. Their antioxidant properties were compared with those of the extract to characterise CQDs synthesised from the plant extract (*E. tenuifolia*). Phenolic molecules in plant extracts contribute to their antioxidant activity and contain multiple–OH groups [64]. Thokchom et al. (2023) reported that the presence of the hydroxyl (–OH) and carboxylate (–COOH) groups in the surface of CQDs can be attributed to their radical scavenging abilities [65]. In the current study, during the carbonisation process, these phenolic molecules with–OH groups were involved in forming CQDs, resulting in the doping of hydroxyl groups on their surface. According to the data from Table 1, the chelation capability of the tested extract (19.87 mg EDTAE/g) was slightly better than that of CQDs (17.93 mg EDTAE/g).

The synthesised CQDs exhibited antioxidant properties comparable to those of the extract. Based on these findings, it was concluded that the antioxidant characteristics of the tested extract were largely maintained throughout the CQD synthesis process. In this sense, the synthesised CQDs can be useful for designing health-promoting applications, including drug delivery.

### Cytotoxicity Tests

**Fig. 7:**
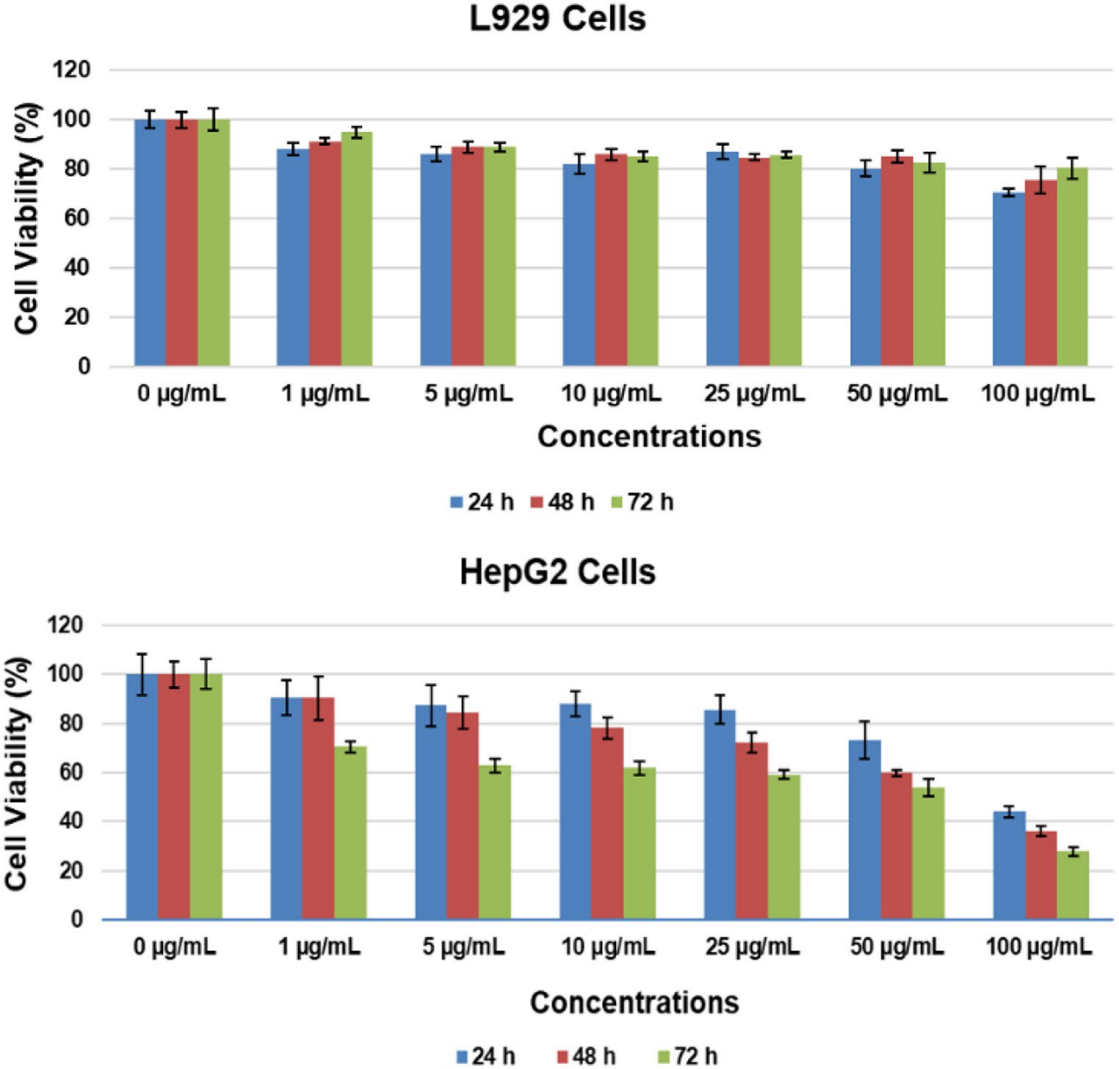
Cytotoxic effect of CQDs on HepG2 and L929 cells

Figure 7 displays a concentration-dependent chart of the cytotoxicity test results conducted over 24, 48, and 72 h. At the end of 24 h, the cell viability of the L929 cell line was calculated to be 70.58 ± 1.38% at 100 µg/mL (***p < 0.001), 80.28 ± 3.31% at 50 µg/mL (***p < 0.001), and 87.14 ± 2.83% at 25 µg/mL (**p < 0.01). All values below 25 µg/mL fell between 82 and 88% (*p < 0.05). At the conclusion of 48 h, the viability values were again calculated in the same order as 75.45 ± 5.36% (***p < 0.001), 85.13 ± 2.42% (***p < 0.001), and 84.74 ± 1.27% (***p < 0.001), and again all values below 25 µg/mL were calculated within the 85–92% range (**p < 0.01). At the end of 72 h, the viability values were calculated to be 80.31 ± 4.03% (***adjusted *p* < 0.001) at 100 µg/mL, 82.62 ± 3.82% (**adjusted *p* < 0.01) at 50 µg/mL, 85.03 ± 0.14% (*adjusted *p* < 0.05) at 10 µg/mL. No statistically significant differences were observed between 1-5-25 µg/mL and control groups (adjusted *p* > 0.05).

The cell viability on the HepG2 cell line was calculated as 73.07 ± 7.67% at 50 µg/mL concentration (**adjusted *p* < 0.01) and 44.05 ± 2.33% (***adjusted *p* < 0.001) at 100 µg/mL after 24 h, respectively. No statistically significant differences were observed between concentrations below 50 µg/mL and control groups (adjusted *p* > 0.05). After 48 h, the calculated viability values for each concentration were 90.31 ± 8.75 (*p* > 0.05), 84.45 ± 6.62 (*p < 0.05), 78.10 ± 4.46 (**p < 0.01), 72.14 ± 3.94 (***p < 0.001), 59.91 ± 1.18 (***p < 0.001), and 36.01 ± 1.94 (***p < 0.001), respectively. At the end of 72 h, there was a dramatic decrease in cell viability and the calculated viability results were as follows: 70.58 ± 2.26 (**p < 0.01), 62.83 ± 2.78 (***p < 0.001), 61.83 ± 2.73 (***p < 0.001), 59.18 ± 1.73 (***p < 0.001), 53.91 ± 3.50 (***p < 0.001), and 27.87 ± 1.76 (***p < 0.001).

According to the ISO 10993-5:2009 standard, in vitro cytotoxicity assessments indicate cytotoxicity when cellular viability percentages fall below 70% (ISO/EN10993-5). Upon examination of the cell viabilities, it was revealed that HepG2 cells exhibited a considerably higher level of cytotoxicity compared to L929 cell lines throughout the initial 24 h-period. The concentration-dependent effect of QDs on cells was also visualised by light microscopy, and the relevant images are presented in Fig. S4. In addition, it is also observed that there is a regular decrease in HEPG2 cells in a time-dependent manner.

Many studies have shown that small-sized nanoparticles (NPs) have a cytotoxic effect at high concentrations [66–70]. The literature reported that quantum dots made of cadmium telluride measuring 2.2 nm were found to be more toxic compared to those measuring 5.5 nm in size [69]. NPs may have a smaller hydrodynamic size, resulting in a larger surface area and increased Cd exposure [71].

The toxicity of QDs depends on the release of metal ions and their stability. In a research study, copper-indium-sulfide QDs without a shell, measuring approximately 3.5 nm, degraded easily in biological fluids. This degradation resulted in in vitro and in vivo toxicity, affecting blood chemistry indexes and the mass of the liver and kidney. However, the presence of a ZnS shell had the potential to alter the toxic properties of these QDs, leading to a significant improvement in their biocompatibility [72]. The toxicity of QDs may vary according to the key parameters of size, concentration, nucleus, coating material, and cell type. To explore the role of core material and surface coatings, namely particle formation in a broad sense, a study was conducted to investigate the toxicity of four types of QDs in two different cancer cell lines (BGC-823-human gastric carcinoma and SH-SY5Y-neuroblastoma) [71]. These formulations commonly used in biological applications are as follows: mercaptopropionic acid-modified CdSe/CdS/ZnS QDs, PEGylated phospholipid encapsulated CdSe/CdS/ZnS and InP/ZnS QDs and Pluronic F127 encapsulated CdTe/ZnS QDs. When BCG-823 cells were used, the CdSe-MPA group showed a concentration-dependent decrease, while cell viability was unaffected in the other groups. Conversely, SH-SYS5Y cells exhibited significant resistance to the CdSe-MPA group, whereas cell viability declined in the remaining groups. The results of that study underscore the complexity of toxicity due to the cellular interactions of QDs (cellular uptake, fate, cell type, and QD formulation, to name a few).

Recently, there has been increasing interest in synthesising QDs using green methods to reduce their toxicity. The cytotoxicity of CQDs derived from *Vitis vinifer*a seeds was determined to be 38.4% against the MCF-7 cell line [73]. Additionally, hemispherical CQDs, with a diameter of 12 nm, synthesised from the leaves of *Hibiscus rosasinensis* Linn. using the microwave-assisted method, exhibited an IC_50_ value of 314 µg/mL against L929 cells [74]. Also, carbon quantum dots (CQDs) obtained from date palm leaves using the hydrothermal technique did not diminish the survival of human mesenchymal stem cells (hMSC) at a level of 200 µg/mL [75]. In another study, six variations of carbon nanodots created through the carbonisation and hydrothermal treatment of plant substances demonstrated no harmful effects on mouse macrophage RAW264.7 cells at a non-hazardous concentration of 2000 µg/mL. They also exhibited minimal toxicity at a concentration of 500 µg/mL when tested on HacaT, MCF-7, and HT-29 cells [76]. The study discovered that even at the highest concentration of CQDs produced from three varieties of bread using frying and hydrothermal methods, over 95% of human colon cancer (HT-29) and mouse colon cancer (CT-26) cells were able to survive [77]. Kamble et al. used the microwave method to extract carbon quantum dots (CQDs) from human hair and then modified them with Poly-L-L-lysine (PLLCQDs) [78]. The PLLCQDs were found to exhibit low cytotoxicity on the fibroblast cell line (L929).

The viability of CQDs (measuring 2–6 nm) produced by the hydrothermal method in the L929 cell line was more than 90% up to 400 mg/mL [79]. It was stated that unaltered carbon dots with positive charges and quaternary ammonium groups reduced cell viability in the L929 cell line while not significantly affecting cell viability in the NIH3/T3 cell line [80]. This revealed that the toxicity of CQDs on mammalian cells varies according to the type of cell, although they are fibroblast cells. Another research indicated that the uptake of L929 and MCF-7 cells was significantly greater compared to NIH/3T3 and HeLa cells [81]. The differences observed in cytotoxicity may be related to the fact that different cell lines have different cellular uptake. Also, antioxidant activity may have a role. A study examined the connection between cytotoxicity and the elimination of free radicals in cancer cells [82]. In our present study, the cytotoxic effect of CQDs differed by concentration and cell type (L929 and HepG2 cells). Also, CQDs are suitable for the size range for cellular uptake. The toxicity of CQDs against different cells can be attributed to their antioxidant activity and cellular uptake.

**Fig. 8:**
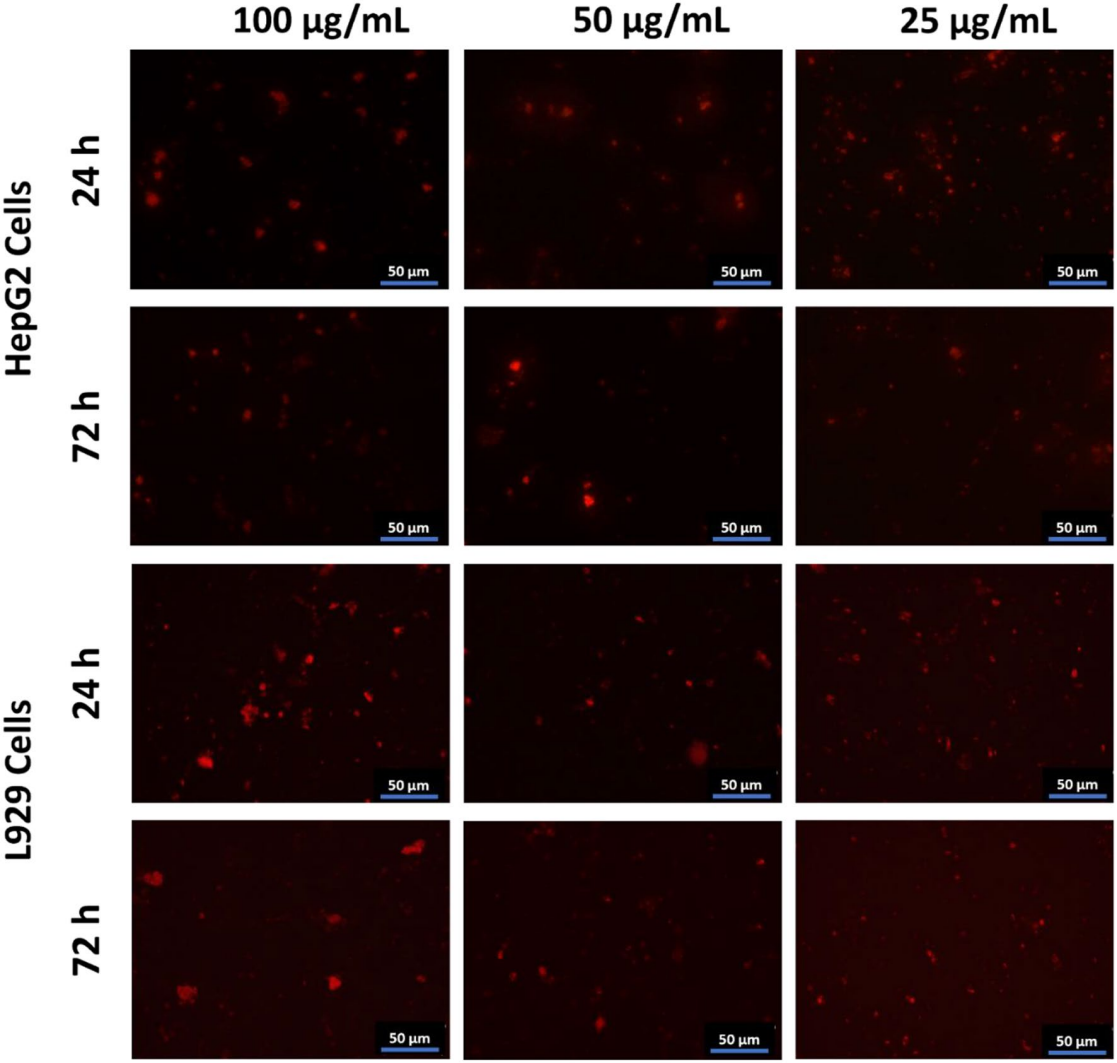
Observation of the effect of the different concentrations of CQDs on fluorescence imaging. Red fluorescence was detected under the Texas red filter

The study investigated how the concentration of CQDs affects imaging, and Fig. 8 shows the corresponding images. Cells treated with CQDs at concentrations lower than 25 µg/mL exhibited no fluorescence.

**Fig. 9:**
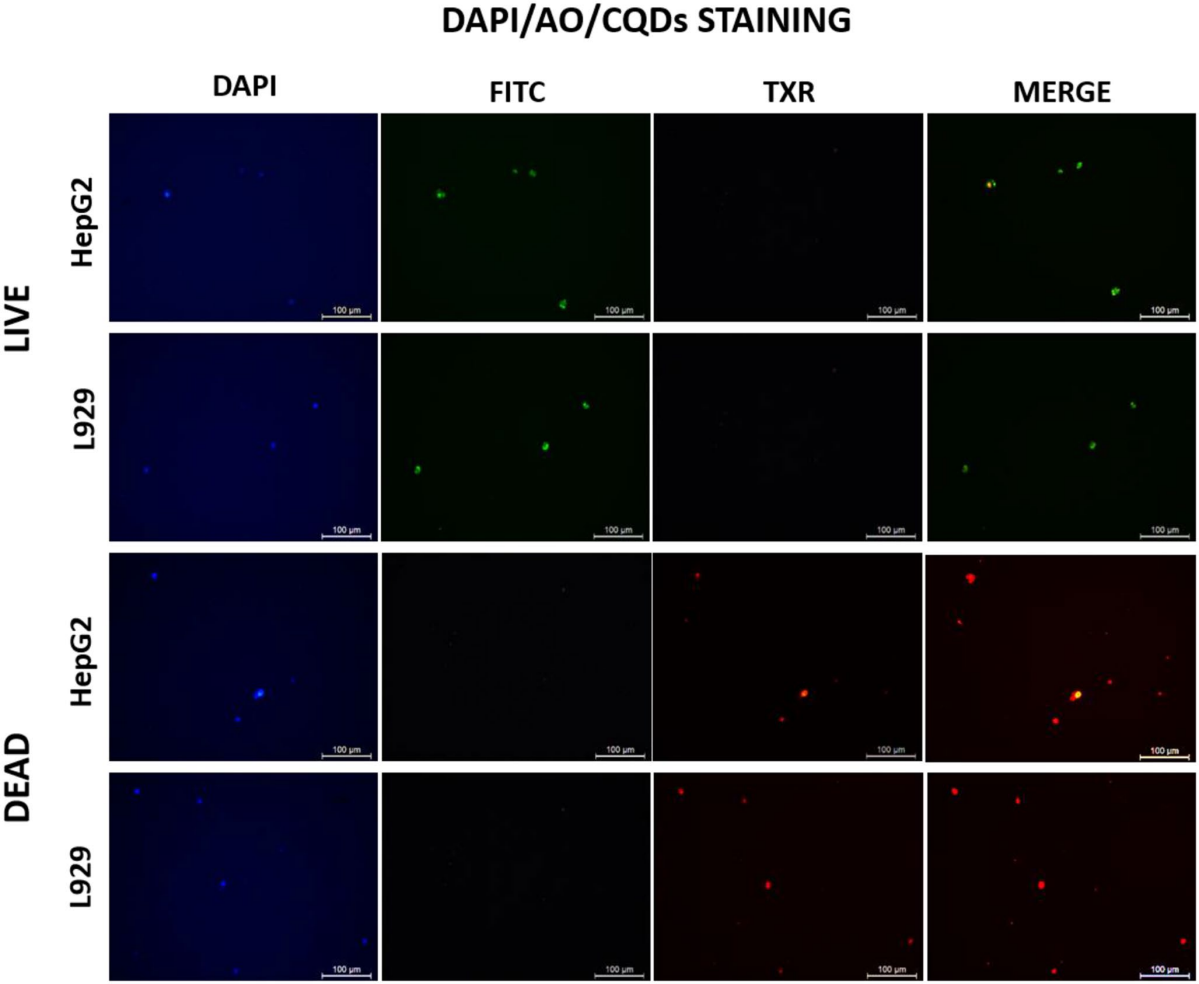
Fluorescence microscopy images of HEPG2 and L929 cell lines

Upon determining the optimal concentration of CQDs as 25 µg/mL, the live and dead staining studies were performed with the help of CQDs and the conventional widely used cell stains such as DAPI, AO and CQDs. The images obtained by a fluorescent microscope containing DAPI, FITC, and Texas Red filter set are given in Fig. 9. As seen in Fig. 9, it was observed that it appeared red colour was under the Texas Red filter. In this study, the cell staining with DAPI/AO/CQDs was performed under the relevant filters, and it was observed that CQDs can stain dead cells. The study results indicate that the ideal concentration for achieving both non-toxicity and fluorescence is 25 µg/mL. Furthermore, it has been found that CQDs can be prepared at a concentration equivalent to other dyes and can be used in combination with them. While the current findings support the selective staining of dead cells by negatively charged CQDs, further validation through differential co-staining with established live/dead dyes is warranted for comprehensive confirmation. Quantum dots exhibit more robust fluorescence brightness and durability than organic fluorescent dyes, and they have a longer fluorescence lifespan. In addition, CQDs are biocompatible when interacting with the cell. According to the research findings, the fluorescence intensity of QDs surpasses that of Rhodamine 6G by a factor of 20 while demonstrating stability 100 times greater than that of Rhodamine 6G [83]. In a different study on this topic, the contrast in the physical and chemical characteristics of traditional organic dyes and QDs was examined and assessed for their potential application as fluorescent markers [84]. Individual QDs were discovered to be 10–20 times more luminous than organic dyes due to their increased light emission rate [83, 85, 86]. Moreover, QDs exhibit greater stability against photodegradation than organic dyes, making them suitable for prolonged monitoring of biological systems [87]. In a study by Dubertret et al. (2002), QDs coated with PEG-derived phospholipid derivatives were introduced into frog embryos for long-term cell imaging and tracking. The results indicated that these QDs were compatible with the biological system and remained stable, supporting normal embryo development for up to 4 days [88].

CQDs are being developed to overcome the shortcomings of fluorescent materials in the field of bioimaging. The use of CQDs has expanded in various fields, such as in vitro and in vivo cell imaging [89, 90], bacterial imaging [91], cancer cell imaging [92] and other fields.

Ji et al. [93] synthesised carbon dots using microwave irradiation for bioimaging of A549 cells and antioxidation applications. The hydrothermal method was used to synthesise nitrogen and sulfur-doped CQDs, which have the potential for in vitro cancer cell imaging [94]. In that study using urea and cysteine as carbon, nitrogen and sulphur sources, the fabricated photoluminescent heteroatom-doped CQDs (UC-CQDs) with a 3.5 nm diameter in size were internalised by cells and emitted green luminescence inside the cells. The normal glial cell line did not experience cell death as a result of the treatment of UC-CQDs. These characteristics showcased how heteroatom-doped UC-CQDs could be used for labelling cells and monitoring their images [94]. Plant-derived CQDs have recently attracted the most attention in in vitro imaging applications. Fluorescent CQDs from the flowers of *Abelmoschus manihot* were tested for detecting 2,4,6-trinitrophenol and for cellular imaging applications [33]. A sensitive fluorescent detection method for microRNA-21 in breast cancer cells was created using a 3D hydrogel made from chitosan and CQDs [95]. N, S self-doped carbon quantum dots (N, S-CQDs) were synthesised from fungus fibres to bioimage cancer cells and sense tetracyclines [96]. In vitro cell imaging applications of N, P-doped CQDs derived from algal biomass (*Dunaliella salina*) were evaluated on HEK-293 cells [97]. CQDs that emit blue light under ultraviolet light have demonstrated fluorescence quenching with high selectivity and sensitivity to harmful metals such as Hg and Cr. In addition, these CQDs were internalised by HEK-293 cells, which resulted in negligible toxicity to them. These results indicate that the fabricated dual-doped CQDs can be used in contaminated living cells for intracellular bioimaging of Hg(II) and Cr(VI). Another study pointed out that 2–3 nm CQDs synthesised from the Pseudo-stem of the banana plant using the hydrothermal method can be used for the intracellular detection of Fe^3+^ in HeLa and MCF-7 cells when treated with 100 µM Fe^3+^ [34].

Colourimetric methods are commonly used to assess cytotoxicity and cell proliferation. However, fluorescent probes may be preferred when evaluating long-term cell viability because they can visualise cells more sensitively. Apoptosis in cancer cells can be determined by different methods [98]. Fluorescence microscopy, one of the morphology-based methods, visualises living and dead cells in various colours using different fluorescent dyes. The critical issue in cell staining is the interaction between the fluorescent dye used for imaging and the cell. While some dyes, such as DAPI, bind specifically to the nucleus, others, such as PI, target the integrity of the cell membrane. Living cells do not allow PI to pass through their intact plasma membranes. However, it can enter dead and apoptotic cells, where it fluoresces strongly. When AO/PI double staining is used to assess apoptosis, AO can enter living cells. Aside from visualising cancer cells, studies were conducted to ascertain if QDs could differentiate between live and deceased cells, similar to fluorescent dye. Four different carbon dots were developed to observe living and dead bacterial cells for this purpose [99]. Nemati et al. documented the cancer-fighting properties of saffron-based CQDs doped with nitrogen and copper (Cu, N-CQDs) against colon cancer cells and their use in bioimaging [100]. They used an AO/PI double staining assay to assess the impact of CQDs on apoptosis, finding that cells exposed to Cu, N-CQDs experienced significant apoptosis. In another study, sulfur-doped organosilica nanodots (S-OSiNDs), which appear orange in colour, have been reported to show selectivity between living and dead cells in staining dead bacterial, fungal and mammalian cells [101]. For discrimination between mammalian cells, S-OSiNDs were found to have a better staining effect than RedDot2 for dead cells.

Chen et al. synthesised silanised carbon quantum dots (Si-CQDs) measuring an average size of 2.8 nm for specific bioimaging of live and dead cells [102]. Dead cells of *S. aureus*, *E. coli*, *Italian penicillium*, and HepG2 were co-stained with a solution of Si-CQDs (20 µg/mL) and PI (20 µg/mL) for 5 min to demonstrate the capability of Si-CQDs in distinguishing between living and deceased cells. The study revealed that Si-CQDs could strongly interact with dead cells but could not fully penetrate the intact cell membranes of viable cells within a brief period. The findings indicate that carbon quantum dots, like fluorescent dyes, have applications in biological imaging. Nonetheless, detailed investigations into the differentiation of living and dead cells are imperative for understanding the underlying mechanism.

In conclusion, *E. tenuifolia*-derived CQDs showing low cytotoxicity and higher fluorescence intensity may also offer selective staining of dead cells. These properties could make CQDs promising alternatives for advanced bioimaging applications, particularly for long-term and selective cellular imaging.

## Conclusion

Fluorescence-based live/dead cell staining is a desirable method to assess cell viability. CQDs are increasingly used to stain live and dead cells. Here, we describe a green synthesis method for creating red-emissive CQDs from the plant *E. tenuifolia*. The in vitro studies showed that the CQDs accumulate differently in live and dead cells, with the latter becoming permeable to the CQDs. They are not toxic to L929 but harmful to HepG2 cells. CQDs have a similar antioxidant capacity to *E. tenuifolia* extract. Red-emissive CQDs can be synthesised from *E. tenuifolia* and used to evaluate bioimaging studies in vitro. Further research is needed to investigate the mechanisms responsible for the uptake of plant-derived CQDs into non-living cells. Future research should focus on understanding the degradation pathways, half-life, and clearance mechanisms of *E. tenuifolia*-derived CQDs to better assess their biocompatibility and long-term behaviour in biological systems.

## Electronic Supplementary Material

Below is the link to the electronic supplementary material.

Supplementary Material 1

## Data Availability

No datasets were generated or analysed during the current study.

## References

[1] Wang B, Cai H, Waterhouse GI, Qu X, Yang B, Lu S (2022) Carbon Dots in bioimaging, biosensing and therapeutics: a comprehensive review. Small Sci 2(6):2200012. 10.1002/smsc.202200012 40213786 10.1002/smsc.202200012 PMC11935858

[2] Konjalwar S, Ceyhan B, Rivera O et al (2024) Demonstrating drug treatment efficacies by monitoring superoxide dynamics in human lung cancer cells with time-lapse fluorescence microscopy. J Biophotonics 17(2):e202300331. 10.1002/jbio.202300331 37822188 10.1002/jbio.202300331 PMC12013861

[3] Metman MJ, Jonker PK, Sondorp LH et al (2024) MET-receptor targeted fluorescent imaging and spectroscopy to detect multifocal papillary thyroid cancer. Eur J Nucl Med Mol Imaging 51(8):2384–2394. 10.1007/s00259-023-06525-5 38017325 10.1007/s00259-023-06525-5 PMC11178647

[4] Mehravanfar H, Farhadian N, Abnous K (2024) Indocyanine green-loaded N-doped carbon quantum Dot nanoparticles for effective photodynamic therapy and cell imaging of melanoma cancer: *In vitro*, ex vivo and *in vivo* study. J Drug Target 32(7):820–837. 10.1080/1061186X.2024.2358511 38779708 10.1080/1061186X.2024.2358511

[5] Schäferling M (2012) The Art of fluorescence imaging with chemical sensors. Angew Chem Int Ed 51:3532–3554. 10.1002/anie.201105459 10.1002/anie.201105459 22422626

[6] Melzer S, Nunes CSM, Endringer DC, De Andrade TU, Tarnok A, Lenz D (2016) Trypan blue as an affordable marker for automated live-dead cell analysis in image cytometry. Scanning 38:857–863. 10.1002/sca.21335 27353800 10.1002/sca.21335

[7] King MA (2000) Detection of dead cells and measurement of cell killing by flow cytometry. J Immunol Methods 243(1–2):155–166. 10.1016/S0022-1759(00)00232-5 10986413 10.1016/s0022-1759(00)00232-5

[8] Gerstner AO, Laffers W, Bootz F, Tarnok A (2003) Use of DNA stains in immunophenotyping by slide-based cytometry. Proc. SPIE 4962, *Manipulation and Analysis of Biomolecules, Cells, and Tissues*, (19 June 2003). 10.1117/12.477892

[9] Hua X-W, Bao Y-W, Wang H-Y, Chen Z, Wu F-G (2017) Bacteria-derived fluorescent carbon Dots for microbial live/dead differentiation. Nanoscale 9(6):2150–2161. 10.1039/C6NR06558A 27874123 10.1039/c6nr06558a

[10] Wegner KD, Hildebrandt N (2015) Quantum Dots: bright and versatile *in vitro* and *in vivo* fluorescence imaging biosensors. Chem Soc Rev 44(14):4792–4834. 10.1039/C4CS00532E 25777768 10.1039/c4cs00532e

[11] Abbigeri MB, Thokchom B, Bhavi SM, Singh SR, Joshi P, Yarajarla RB (2024) Potential *in vitro* antibacterial and anticancer properties of biosynthesized multifunctional silver nanoparticles using *Martynia annua* L. leaf extract. Nano-Structures Nano-Objects 39:101320. 10.1016/j.nanoso.2024.101320

[12] Bhavi SM, Thokchom B, Singh SR, Bajire SK, Shastry RP, Srinath BS et al (2025) *Syzygium malaccense* leaf extract-mediated silver nanoparticles: synthesis, characterization, and biomedical evaluation in *Caenorhabditis elegans* and lung cancer cell line. Green Chem Lett Rev 18(1):2456624. 10.1080/17518253.2025.2456624

[13] Manikandan V, Lee NY (2022) Green synthesis of carbon quantum Dots and their environmental applications. Environ Res 212:113283. 10.1016/j.envres.2022.113283 35461844 10.1016/j.envres.2022.113283

[14] Liu J, Li R, Yang B (2020) Carbon Dots: A new type of carbon-based nanomaterial with wide applications. ACS Cent Sci 6(12):2179–2195. 10.1021/acscentsci.0c01306 33376780 10.1021/acscentsci.0c01306 PMC7760469

[15] Olmos-Moya PM, Velazquez-Martinez S, Pineda-Arellano C, Rangel-Mendez JR, Chazaro-Ruiz LF (2022) High added value functionalized carbon quantum Dots synthetized from orange peels by assisted microwave solvothermal method and their performance as photosensitizer of mesoporous TiO_2_ photoelectrodes. Carbon 187:216–229. 10.1016/j.carbon.2021.11.003

[16] Wu S, Fang L, Li Y, Wang H-B, Zhang H (2023) A fluorescence turn on–off-on method for sensitive detection of Sn^2+^ and Glycine using waste eggshell membrane derived carbon nanodots as probe. J Fluoresc 33:1505–1513. 10.1007/s10895-022-03133-8 36763295 10.1007/s10895-022-03133-8

[17] Zhang L, Li B, Zhou Y, Wu Y, Le T, Sun Q (2023) Green synthesis of cow milk-derived carbon quantum Dots and application for Fe^3+^ detection. J Sol-Gel Sci Technol 106:173–185. 10.1007/s10971-022-06024-3

[18] Min S, Ezati P, Yoon KS, Rhim J-W (2023) Gelatin/poly (vinyl alcohol)-based functional films integrated with spent coffee ground-derived carbon Dots and grapefruit seed extract for active packaging application. Int J Biol Macromol 231:123493. 10.1016/j.ijbiomac.2023.123493 36731691 10.1016/j.ijbiomac.2023.123493

[19] Arumugham T, Alagumuthu M, Amimodu RG, Munusamy S, Iyer SK (2020) A sustainable synthesis of green carbon quantum Dot (CQD) from *Catharanthus roseus* (white flowering plant) leaves and investigation of its dual fluorescence responsive behavior in multi-ion detection and biological applications. Sustain Mater Technol 23:e00138. 10.1016/j.susmat.2019.e00138

[20] Nasseri MA, Keshtkar H, Kazemnejadi M, Allahresani A (2020) Phytochemical properties and antioxidant activity of *Echinops persicus* plant extract: green synthesis of carbon quantum Dots from the plant extract. SN Appl Sci 2:670. 10.1007/s42452-020-2466-0

[21] Lin L, Xia Y, Wen H et al (2023) Green and continuous microflow synthesis of fluorescent carbon quantum Dots for bio-imaging application. AIChE J 69(1):e17901. 10.1002/aic.17901

[22] Han G, Zhao J, Zhang R et al (2019) Membrane-penetrating carbon quantum Dots for imaging nucleic acid structures in live organisms. Angew Chem 131(21):7161–7165. 10.1002/ange.201903005 10.1002/anie.201903005 30912239

[23] Zhang Y, Liu X, Fan Y et al (2016) One-step microwave synthesis of N-doped hydroxyl-functionalized carbon Dots with ultra-high fluorescence quantum yields. Nanoscale 8(33):15281–15287. 10.1039/C6NR03125K 27500530 10.1039/c6nr03125k

[24] Zhu S, Meng Q, Wang L et al (2013) Highly photoluminescent carbon Dots for multicolor patterning, sensors, and bioimaging. Angew Chem Int Ed 52:3953–3957. 10.1002/anie.201300519 10.1002/anie.201300519 23450679

[25] Ding H, Yu S-B, Wei J-S, Xiong H-M (2016) Full-color light-emitting carbon Dots with a surface-state-controlled luminescence mechanism. ACS Nano 10(1):484–491. 10.1021/acsnano.5b05406 26646584 10.1021/acsnano.5b05406

[26] Nie H, Li M, Li Q et al (2014) Carbon Dots with continuously tunable full-color emission and their application in ratiometric pH sensing. Chem Mater 26(10):3104–3112. 10.1021/cm5003669

[27] Liu J, Li D, Zhang K, Yang M, Sun H, Yang B (2018) One-step hydrothermal synthesis of nitrogen‐doped conjugated carbonized polymer Dots with 31% efficient red emission for *in vivo* imaging. Small 14(15):1703919. 10.1002/smll.201703919 10.1002/smll.201703919 29508542

[28] Lu S, Sui L, Liu J et al (2017) Near-Infrared photoluminescent polymer–carbon nanodots with two-photon fluorescence. Adv Mater 29(15):1603443. 10.1002/adma.201603443 10.1002/adma.201603443 28195369

[29] Liu J, Geng Y, Li D et al (2020) Deep red emissive carbonized polymer Dots with unprecedented narrow full width at half maximum. Adv Mater 32(17):1906641. 10.1002/adma.201906641 10.1002/adma.201906641 32191372

[30] Zhao F, Li X, Zuo M et al (2023) Preparation of photocatalysts decorated by carbon quantum Dots (CQDs) and their applications: A review. J Environ Chem Eng 11(2):109487. 10.1016/j.jece.2023.109487

[31] Desmond LJ, Phan AN, Gentile P (2021) Critical overview on the green synthesis of carbon quantum Dots and their application for cancer therapy. Environ Sci Nano 8(4):848–862. 10.1039/D1EN00017A

[32] Bressi V, Balu AM, Iannazzo D, Espro C (2022) Recent advances in the synthesis of carbon Dots from renewable biomass by high-efficient hydrothermal and microwave green approaches. Curr Opin Green Sustain Chem 40:100742. 10.1016/j.cogsc.2022.100742

[33] Wan Y, Wang M, Zhang K et al (2019) Facile and green synthesis of fluorescent carbon Dots from the flowers of Abelmoschus manihot (Linn.) medicus for sensitive detection of 2, 4, 6-trinitrophenol and cellular imaging. Microchem J 148:385–396. 10.1016/j.microc.2019.05.026

[34] Vandarkuzhali SAA, Jeyalakshmi V, Sivaraman G, Singaravadivel S, Krishnamurthy KR, Viswanathan B (2017) Highly fluorescent carbon Dots from pseudo-stem of banana plant: applications as nanosensor and bio-imaging agents. Sens Actuators B Chem 252:894–900. 10.1016/j.snb.2017.06.088

[35] Gokbulut I, Bilenler T, Karabulut I (2013) Determination of chemical composition, total phenolic, antimicrobial, and antioxidant activities of *Echinophora tenuifolia* essential oil. Int J Food Proper 167:1442–1451. 10.1080/10942912.2011.593281

[36] Ivanova S, Dyankov S, Karcheva-Bahchevanska D et al (2023) *Echinophora tenuifolia* subsp. sibthorpiana—Study of the histochemical localization of essential oil. Molecules 28(7):2918. 10.3390/molecules28072918 37049678 10.3390/molecules28072918 PMC10096146

[37] Sargin I, Karakurt S, Alkan S, Arslan G (2021) Live cell imaging with biocompatible fluorescent carbon quantum Dots derived from edible mushrooms *Agaricus bisporus*, *Pleurotus ostreatus*, and *Suillus luteus*. J Fluoresc 31:1461–1473. 10.1007/s10895-021-02784-3 34279764 10.1007/s10895-021-02784-3

[38] Mosmann T (1983) Rapid colorimetric assay for cellular growth and survival: application to proliferation and cytotoxicity assays. J Immunol Methods 65(1–2):55–63. 10.1016/0022-1759(83)90303-4 6606682 10.1016/0022-1759(83)90303-4

[39] Uysal S, Zengin G, Locatelli M et al (2017) Cytotoxic and enzyme inhibitory potential of two potentilla species *(P. speciosa* L. and *P. reptans* Willd.) and their chemical composition. Front Pharmacol 8:290. 10.3389/fphar.2017.00290 28588492 10.3389/fphar.2017.00290 PMC5441381

[40] Wang Y, Hu A (2014) Carbon quantum Dots: synthesis, properties and applications. J Mater Chem C 2(34):6921–6939. 10.1039/C4TC00988F

[41] Bao L, Zhang ZL, Tian ZQ et al (2011) Electrochemical tuning of luminescent carbon nanodots: from Preparation to luminescence mechanism. Adv Mater 23(48):5801–5806. 10.1002/adma.201102866 22144369 10.1002/adma.201102866

[42] Li H, He X, Kang Z et al (2010) Water-soluble fluorescent carbon quantum Dots and photocatalyst design. Angew Chem 122:4532–4536. 10.1002/ange.200906154 10.1002/anie.200906154 20461744

[43] Das R, Bandyopadhyay R, Pramanik P (2018) Carbon quantum Dots from natural resource: A review. Mater Today Chem 8:96–109. 10.1016/j.mtchem.2018.03.003

[44] Alam MB, Minocha T, Yadav SK, Parmar AS (2022) Therapeutic potential of chlorophyll functionalized carbon quantum Dots against cervical cancer. ChemistrySelect 7(48):e202204562. 10.1002/slct.202204562

[45] Swapna M, Raj V, Devi HS, Sankararaman S (2019) Optical emission diagnosis of carbon nanoparticle-incorporated chlorophyll for sensing applications. Photochem Photobiol Sci 18:1382–1388. 10.1039/c8pp00454d 30919854 10.1039/c8pp00454d

[46] Cheng B, Cao L, Li C et al (2023) Fluorine-doped carbon quantum Dots with deep-red emission for hypochlorite determination and cancer cell imaging. Chin Chem Lett 108969. 10.1016/j.cclet.2023.108969

[47] Arumugam N, Kim J (2018) Synthesis of carbon quantum Dots from broccoli and their ability to detect silver ions. Mater Lett 219:37–40. 10.1016/j.matlet.2018.02.043

[48] De B, Karak N (2013) A green and facile approach for the synthesis of water soluble fluorescent carbon Dots from banana juice. RSC Adv 3(22):8286–8290. 10.1039/C3RA00088E

[49] Nishino M, Matsuzaki I, Musangile FY et al (2020) Measurement and visualization of cell membrane surface charge in fixed cultured cells related with cell morphology. PLoS ONE 15(7):e0236373. 10.1371/journal.pone.0236373 32702063 10.1371/journal.pone.0236373 PMC7377470

[50] Šafranko S, Goman D, Stanković A et al (2021) An overview of the recent developments in carbon quantum dots—promising nanomaterials for metal ion detection and (bio) molecule sensing. Chemosensors 9(6):138. 10.3390/chemosensors9060138

[51] Thambiraj S, Shankaran R (2016) Green synthesis of highly fluorescent carbon quantum Dots from sugarcane Bagasse pulp. Appl Surf Sci 390:435–443. 10.1016/j.apsusc.2016.08.106

[52] Huang X, Yang L, Hao S et al (2017) N-Doped carbon Dots: a metal-free co-catalyst on hematite Nanorod arrays toward efficient photoelectrochemical water oxidation. Inorg Chem Front 4(3):537–540. 10.1039/C6QI00517A

[53] Ferjani H, Abdalla S, Oyewo OA, Onwudiwe DC (2024) Facile synthesis of carbon Dots by the hydrothermal carbonization of avocado peels and evaluation of the photocatalytic property. Inorg Chem Commun 160:111866. 10.1016/j.inoche.2023.111866

[54] Bibi Sadeer N, Montesano D, Albrizio S, Zengin G, Mahomoodally MF (2020) The versatility of antioxidant assays in food science and safety—Chemistry, applications, strengths, and limitations. Antioxidants 9(8):709. 10.3390/antiox9080709 32764410 10.3390/antiox9080709 PMC7464350

[55] Rodríguez-Varillas S, Fontanil T, Obaya ÁJ, Fernández-González A, Murru C, Badía-Laíño R (2022) Biocompatibility and antioxidant capabilities of carbon Dots obtained from tomato (*Solanum lycopersicum*). Appl Sci 12(2):773. 10.3390/app12020773

[56] Gedda G, Sankaranarayanan SA, Putta CL, Gudimella KK, Rengan AK, Girma WM (2023) Green synthesis of multi-functional carbon Dots from medicinal plant leaves for antimicrobial, antioxidant, and bioimaging applications. Sci Rep 13(1):6371. 10.1038/s41598-023-33652-8 37076562 10.1038/s41598-023-33652-8 PMC10115846

[57] Innocenzi P, Stagi L (2023) Carbon Dots as oxidant-antioxidant nanomaterials, Understanding the structure-properties relationship. A critical review. Nano Today 50:101837. 10.1016/j.nantod.2023.101837

[58] Koutamehr ME, Moradi M, Tajik H, Molaei R, Heshmati MK, Alizadeh A (2023) Sour whey-derived carbon dots; synthesis, characterization, antioxidant activity and antimicrobial performance on foodborne pathogens. LWT 184:114978. 10.1016/j.lwt.2023.114978

[59] Li Q, Shen X, Xing D (2023) Carbon quantum Dots as ROS-generator and-scavenger: A comprehensive review. Dyes Pigm 208:110784. 10.1016/j.dyepig.2022.110784

[60] Choi SS, Park HR, Lee KA (2021) A comparative study of Rutin and Rutin glycoside: antioxidant activity, anti-inflammatory effect, effect on platelet aggregation and blood coagulation. Antioxidants 10(11):1696. 10.3390/antiox10111696 34829567 10.3390/antiox10111696 PMC8614652

[61] Wang L, Pan X, Jiang L et al (2022) The biological activity mechanism of chlorogenic acid and its applications in food industry: A review. Front Nutr 9:943911. 10.3389/fnut.2022.943911 35845802 10.3389/fnut.2022.943911 PMC9278960

[62] Enogieru AB, Haylett W, Hiss DC, Bardien S, Ekpo OE (2018) Rutin as a potent antioxidant: implications for neurodegenerative disorders. Oxid Med Cell Longev 2018:6241017. 10.1155/2018/6241017 30050657 10.1155/2018/6241017 PMC6040293

[63] Sato Y, Itagaki S, Kurokawa T et al (2011) In vitro and in vivo antioxidant properties of chlorogenic acid and caffeic acid. Int J Pharm 403(1–2):136–138. 10.1016/j.ijpharm.2010.09.035 20933071 10.1016/j.ijpharm.2010.09.035

[64] Alara OR, Abdurahman NH, Ukaegbu CI (2021) Extraction of phenolic compounds: A review. Curr Res Food Sci 4:200–214. 10.1016/j.crfs.2021.03.011 33899007 10.1016/j.crfs.2021.03.011 PMC8058613

[65] Thokchom B, Bhavi SM, Abbigeri MB et al (2023) Green synthesis, characterization and biomedical applications of *Centella asiatica*-derived carbon Dots. Carbon Lett 33:1057–1071. 10.1007/s42823-023-00505-3

[66] Lovrić J, Bazzi HS, Cuie Y, Fortin GR, Winnik FM, Maysinger D (2005) Differences in subcellular distribution and toxicity of green and red emitting CdTe quantum Dots. J Mol Med 83:377–385. 10.1007/s00109-004-0629-x 15688234 10.1007/s00109-004-0629-x

[67] Munari M, Sturve J, Frenzilli G et al (2014) Genotoxic effects of cds quantum Dots and Ag_2_S nanoparticles in fish cell lines (RTG-2). Mutat Res Genet Toxicol Environ Mutagen 775–776:89–93. 10.1016/j.mrgentox.2014.09.003 25435359 10.1016/j.mrgentox.2014.09.003

[68] Zhang Y, Chen W, Zhang J, Liu J, Chen G, Pope C (2007) *In vitro* and *in vivo* toxicity of CdTe nanoparticles. J Nanoscie Nanotechnol 7(2):497–503. 10.1166/jnn.2007.125 10.1166/jnn.2007.125 17450785

[69] Lovrić J, Cho SJ, Winnik FM, Maysinger D (2005) Unmodified cadmium telluride quantum Dots induce reactive oxygen species formation leading to multiple organelle damage and cell death. Chem Biol 12(11):1227–1234. 10.1016/j.chembiol.2005.09.008 16298302 10.1016/j.chembiol.2005.09.008

[70] Wiesner MR, Lowry GV, Alvarez P, Dionysiou D, Biswas P (2006) Assessing the risks of manufactured nanomaterials. Environ Sci Technol 40(14):4336–4345. 10.1021/es062726m 16903268 10.1021/es062726m

[71] Liu J, Hu R, Liu J et al (2015) Cytotoxicity assessment of functionalized CdSe, CdTe and inp quantum Dots in two human cancer cell models. Mater Sci Eng C 57:222–231. 10.1016/j.msec.2015.07.044 10.1016/j.msec.2015.07.044 26354258

[72] Kays JC, Saeboe AM, Toufanian R, Kurant DE, Dennis AM (2020) Shell-free copper indium sulfide quantum Dots induce toxicity *in vitro* and *in vivo*. Nano Lett 20(3):1980–1991. 10.1021/acs.nanolett.9b05259 31999467 10.1021/acs.nanolett.9b05259 PMC7210713

[73] Parvathy C, Praseetha P (2023) Carbon quantum Dot induced hemolysis and anti-angiogenesis in proliferating cancers with *Vitis vinifera* as the source material. Vegetos 36(3):890–898. 10.1007/s42535-022-00454-8

[74] Yalshetti S, Thokchom B, Bhavi SM et al (2024) Microwave-assisted synthesis, characterization and *in vitro* biomedical applications of *Hibiscus rosa-sinensis* Linn.-mediated carbon quantum Dots. Sci Rep 14(1):9915. 10.1038/s41598-024-60726-y 38689005 10.1038/s41598-024-60726-y PMC11061284

[75] Athinarayanan J, Periasamy VS, Al-Harbi LN, Alshatwi AA (2023) *Phoenix dactylifera* leaf-derived biocompatible carbon quantum Dots: application in cell imaging. Biomass Conv Bioref 13(14):12989–12998. 10.1007/s13399-021-02159-5

[76] Chen Y-Y, Jiang W-P, Chen H-L et al (2021) Cytotoxicity and cell imaging of six types of carbon nanodots prepared through carbonization and hydrothermal processing of natural plant materials. RSC Adv 11(27):16661–16674. 10.1039/D1RA01318A 35479143 10.1039/d1ra01318a PMC9031421

[77] Anpalagan K, Karakkat JV, Jelinek R et al (2023) A green synthesis route to derive carbon quantum Dots for bioimaging cancer cells. Nanomaterials 13(14):2103. 10.3390/nano13142103 37513114 10.3390/nano13142103 PMC10385789

[78] Kamble P, Malavekar D, Tiwari AP (2024) Natural Biowaste derived fluorescent carbon quantum Dots: synthesis, characterization and biocompatibility study. J Fluoresc 34:191–201. 10.1007/s10895-023-03244-w 37166612 10.1007/s10895-023-03244-w

[79] Huang C, Dong H, Su Y, Wu Y, Narron R, Yong Q (2019) Synthesis of carbon quantum Dot nanoparticles derived from byproducts in bio-refinery process for cell imaging and *in vivo* bioimaging. Nanomaterials 9(3):387. 10.3390/nano9030387 30866423 10.3390/nano9030387 PMC6473984

[80] Havrdová M, Urbančič I, Bartoň Tománková K, Malina L, Štrancar J, Bourlinos AB (2021) Self-targeting of carbon Dots into the cell nucleus: diverse mechanisms of toxicity in NIH/3T3 and L929 cells. Int J Mol Sci 22(11):5608. 10.3390/ijms22115608 34070594 10.3390/ijms22115608 PMC8198156

[81] Havrdová M, Urbančič I, Tománková KB et al (2022) Intracellular trafficking of cationic carbon Dots in cancer cell lines MCF-7 and HeLa—time lapse microscopy, concentration-dependent uptake, viability, DNA damage, and cell cycle profile. Int J Mol Sci 23(3):1077. 10.3390/ijms23031077 35162996 10.3390/ijms23031077 PMC8835431

[82] Sammar M, Abu–Farich B, Rayan I, Falah M, Rayan A (2019) Correlation between cytotoxicity in cancer cells and free radical–scavenging activity: *In vitro* evaluation of 57 medicinal and edible plant extracts. Oncol Lett 18(6):6563–6571. 10.3892/ol.2019.11054 31819777 10.3892/ol.2019.11054 PMC6896308

[83] Gao X, Yang L, Petros JA, Marshall FF, Simons JW, Nie S (2005) *In vivo* molecular and cellular imaging with quantum Dots. Curr Opin Biotechnol 16(1):63–72. 10.1016/j.copbio.2004.11.003 15722017 10.1016/j.copbio.2004.11.003

[84] Resch-Genger U, Grabolle M, Cavaliere-Jaricot S, Nitschke R, Nann T (2008) Quantum Dots versus organic dyes as fluorescent labels. Nat Methods 5(9):763–775. 10.1038/nmeth.1248 18756197 10.1038/nmeth.1248

[85] Chan WC, Nie S (1998) Quantum Dot bioconjugates for ultrasensitive nonisotopic detection. Science 281(5385):2016–2018. 10.1126/science.281.5385.2016 9748158 10.1126/science.281.5385.2016

[86] Bruchez M Jr, Moronne M, Gin P, Weiss S, Alivisatos AP (1998) Semiconductor nanocrystals as fluorescent biological labels. Science 281(5385):2013–2016. 10.1126/science.281.5385.201 9748157 10.1126/science.281.5385.2013

[87] Smith AM, Dave S, Nie S, True L, Gao X (2006) Multicolor quantum Dots for molecular diagnostics of cancer. Expert Rev Mol Diagn 6(2):231–244. 10.1586/14737159.6.2.231 16512782 10.1586/14737159.6.2.231

[88] Dubertret B, Skourides P, Norris DJ, Noireaux V, Brivanlou AH, Libchaber A (2002) *In vivo* imaging of quantum Dots encapsulated in phospholipid micelles. Science 298(5599):1759–1762. 10.1126/science.1077194 12459582 10.1126/science.1077194

[89] Liu Y, Song Y, Zhang J et al (2021) Responsive carbonized polymer Dots for optical super-resolution and fluorescence lifetime imaging of nucleic acids in living cells. ACS Appl Mater Interfaces 13(43):50733–50743. 10.1021/acsami.1c13943 34670368 10.1021/acsami.1c13943

[90] Singh V, Rawat KS, Mishra S et al (2018) Biocompatible fluorescent carbon quantum dots prepared from beetroot extract for *in vivo* live imaging in *C. elegans* and BALB/c mice. *J Mater Chem B* 6(20):3366–3371. 10.1039/C8TB00503F 10.1039/c8tb00503f 32254394

[91] Sharma N, Sharma I, Bera MK (2022) Microwave-assisted green synthesis of carbon quantum Dots derived from *Calotropis gigantea* as a fluorescent probe for bioimaging. J Fluoresc 32(3):1039–1049. 10.1007/s10895-022-02923-4 35262854 10.1007/s10895-022-02923-4

[92] Bajpai VK, Khan I, Shukla S et al (2020) Multifunctional NP-doped carbon Dots for regulation of apoptosis and autophagy in B16F10 melanoma cancer cells and *in vitro* imaging applications. Theranostics 10(17):7841–7856. 10.7150/thno.42291 32685024 10.7150/thno.42291 PMC7359102

[93] Ji Z, Yin Z, Jia Z, Wei J (2020) Carbon nanodots derived from Urea and citric acid in living cells: cellular uptake and antioxidation effect. Langmuir 36(29):8632–8640. 10.1021/acs.langmuir.0c01598 32610019 10.1021/acs.langmuir.0c01598

[94] Ghosh T, Nandi S, Bhattacharyya SK et al (2023) Nitrogen and sulphur doped carbon Dot: an excellent biocompatible candidate for in-vitro cancer cell imaging and beyond. Environ Res 217:114922. 10.1016/j.envres.2022.114922 36435492 10.1016/j.envres.2022.114922

[95] Mohammadi S, Mohammadi S, Salimi A (2021) A 3D hydrogel based on Chitosan and carbon Dots for sensitive fluorescence detection of microRNA-21 in breast cancer cells. Talanta 224:121895. 10.1016/j.talanta.2020.121895 33379103 10.1016/j.talanta.2020.121895

[96] Shi C, Qi H, Ma R et al (2019) N, S-self-doped carbon quantum Dots from fungus fibers for sensing tetracyclines and for bioimaging cancer cells. Mater Sci Eng C 105:110132. 10.1016/j.msec.2019.110132 10.1016/j.msec.2019.110132 31546396

[97] Singh AK, Singh VK, Singh M et al (2019) One pot hydrothermal synthesis of fluorescent NP-carbon Dots derived from Dunaliella Salina biomass and its application in on-off sensing of hg (II), cr (VI) and live cell imaging. J Photochem Photobiol A 376:63–72. 10.1016/j.jphotochem.2019.02.023

[98] Ulukaya E, Acilan C, Ari F, Ikitimur E, Yilmaz Y (2011) A glance at the methods for detection of apoptosis qualitatively and quantitatively. Turk J Biochem 36(3):261–269

[99] Liu Y, Zhong D, Yu L, Shi Y, Xu Y (2023) Primary amine functionalized carbon Dots for dead and alive bacterial imaging. Nanomaterials 13(3):437. 10.3390/nano13030437 36770398 10.3390/nano13030437 PMC9920602

[100] Nemati M, Hallaj T, Rezaie J, Rasmi Y (2023) Nitrogen and copper-doped saffron-based carbon Dots: synthesis, characterization, and cytotoxic effects on human colorectal cancer cells. Life Sci 319:121510. 10.1016/j.lfs.2023.121510 36813083 10.1016/j.lfs.2023.121510

[101] Li Y-H, Zeng J, Wang Z et al (2022) Sulfur-doped Organosilica nanodots as a universal sensor for ultrafast live/dead cell discrimination. Biosensors 12(11):1000. 10.3390/bios12111000 36354509 10.3390/bios12111000 PMC9688158

[102] Chen J, Liu W-R, Li Y et al (2022) Architecting ultra-bright silanized carbon Dots by alleviating the spin-orbit coupling effect: A specific fluorescent nanoprobe to label dead cells. Chem Eng J 428:131168. 10.1016/j.cej.2021.131168

