# Supplementary material for: Dead Cell Discrimination with Red Emissive Carbon Quantum Dots from the Medicinal and Edible Herb Echinophora tenuifolia
Source: J Fluoresc. 2025 Apr 5;35(10):9889–906. doi: 10.1007/s10895-025-04286-y (PMC12672788; doi:10.1007/s10895-025-04286-y)
Supplement: Supplementary file 1 — Supplementary Material 1 [file 10895_2025_4286_MOESM1_ESM.docx]

**Supporting Information**

# Dead cell discrimination with red emissive carbon quantum dots from the medicinal and edible herb *Echinophora tenuifolia*

**1. Instrumentation**

Morphological, structural and photophysical analysis of CQDs was performed using the following analytical tools. Transmission electron microscopy (TEM) images of CQDs were recorded on JEOL JEM-2100, UHR–Ultra High-Resolution device. X-ray diffractograms (XRD) of CQDs were obtained using a Bruker D8 Advance X-ray diffractometer; The 2θ angle was scanned between 0° and 90° at a scanning speed of 3° min^–1^. Fourier transform infrared (FT–IR) spectra of CQDs were recorded on a Bruker Vertex 70 FT-IR spectrometer in the range of 4000 and 500 cm^–1^. X-ray Photoelectron Spectroscopy was used to obtain XPS scans using the Thermo Scientific K-Alpha X-ray Photoelectron Spectrometer (Number of Scans: 10, Source Gun Type: Al K Alpha, Spot size: 400 µm, lens mode: standard, analyser mode: CAE, pass energy: 30.0 eV, energy step size: 0.100 eV). Ultraviolet-visible (UV–vis) absorbance spectra of CQDs were obtained using Shimadzu UV1800 UV–vis absorption spectrophotometer. Fluorescence spectra of CQDs were obtained using the Perkin Elmer LS 55 instrument, Cambridge, UK. CQD synthesis was performed in a microwave digestion oven (CEM Mars 5). Zeta potential measurements of the CQDs dispersed in water were performed (Malvern Zetasizer Nano ZS90).

**2. Optimisation of CQDs synthesis**

|  |  |
| --- | --- |
|  |  |

**Fig. S1** Four reaction parameters (upper left to right: microwave exposure time, solvent type, lower left to right: microwave power, and plant amount) were tested to optimise the synthesis of CQDs from the plant *E. tenuifolia*.

**2. Fluorescence quantum yield of CQDs**

The absorbance of quinine sulfate and CQDs solutions was recorded as their concentrations increased: quinine sulfate in 0.1 M H_2_SO_4_ (refractive index η = 1.33) and CQDs in distilled water (η = 1.33). To avoid re-absorption effects, the absorbance for both solutions was kept below 0.1 at wavelengths of 320 nm and above. Fluorescence emission spectra were captured using a fluorescence spectrometer, with an excitation wavelength of 320 nm. The integrated fluorescence emission from these solutions was derived from the emission spectra within the range of 345-595 nm. The integrated fluorescence emission intensity was then plotted against the relevant UV-vis absorbance values (intercept = 0).

**3. HPLC analysis of the extracts**

Phenolic compounds were evaluated by reversed-phase high-performance liquid chromatography (RP-HPLC, Shimadzu Scientiﬁc Instruments, Kyoto, Japan). Detection and quantiﬁcation were carried out with an LC-10ADvp pump, a diode array detector, a CTO-10Avp column heater, an SCL-10Avp system controller, DGU-14A degasser and SIL-10ADvp autosampler (Shimadzu Scientiﬁc Instruments, Columbia, MD, USA). Separations were conducted at 30°C on Agilent® Eclipse XDB C-18 reversed-phase column (250 mm x4.6 mm length, 5 µm particle size, Agilent, Santa Clara, CA, USA). The eluates were detected at 278 nm. The mobile phases were A: 3.0% acetic acid in distilled water and B: methanol. The samples were dissolved in methanol for analysis, and 20 µL of this solution was injected into the column. The elution gradient applied at a flow rate of 0.8 ml/min was 93% A/7% B for 0.1 min, 72%A/28%B in 20 min, 75%A/25%B in 8 min, 70%A/30%B in 7 min and same gradient for 15 min, 67%A/33%B in 10 min, 58%A/42%B in 2 min, 50%A/50%B in 8 min, 30%A/70%B in 3 min, 20%A/80%B in 2 min 100%B in 5 min until the end of the run. Identification and quantitative analysis were carried out by comparison with standards. Each phenolic compound was expressed in g per gram of extract using external calibration curves obtained for each phenolic standard at corresponding absorption maxima for specific classes of phenols (i.e., phenolic acids and flavonoids). Commercial standards were prepared in methanol.


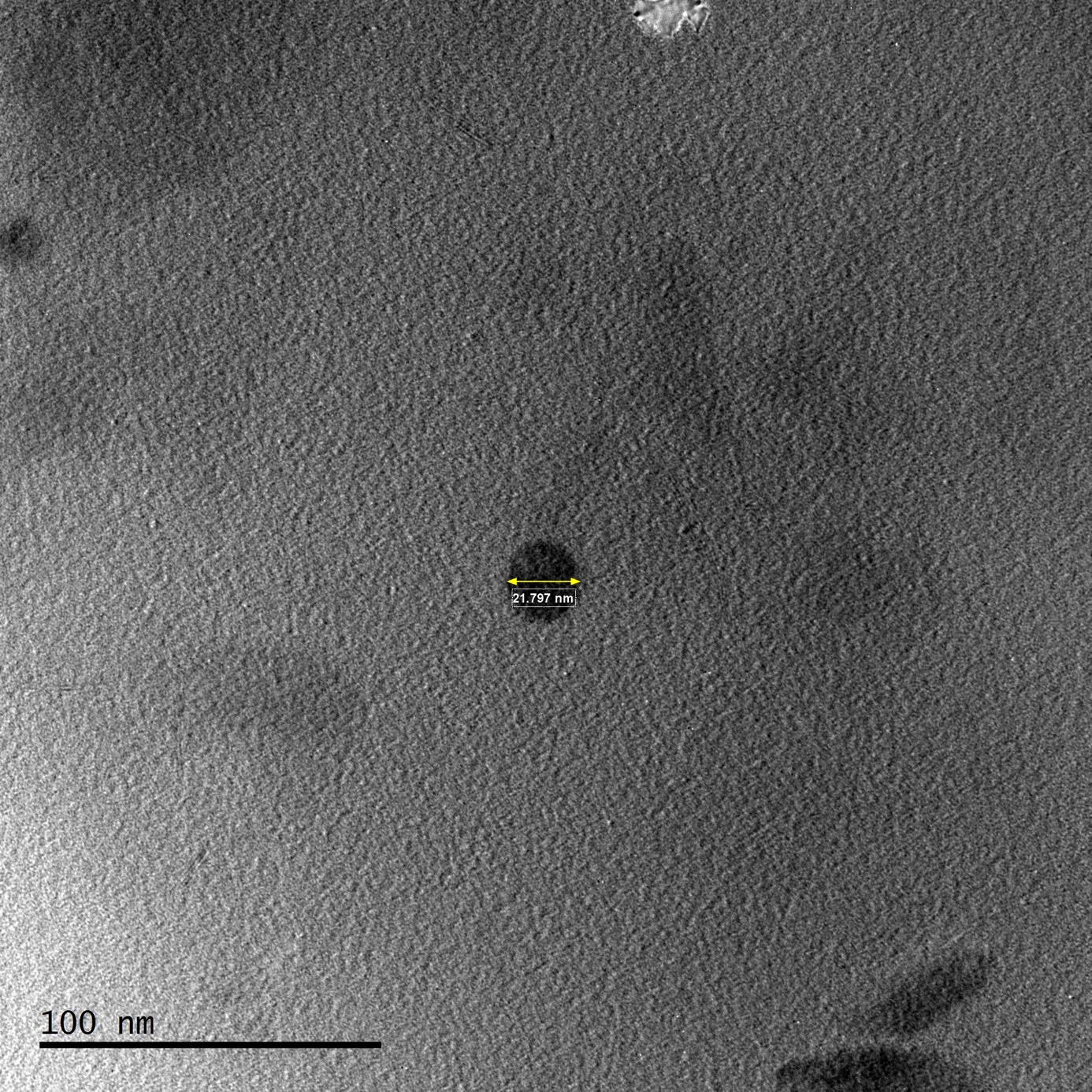

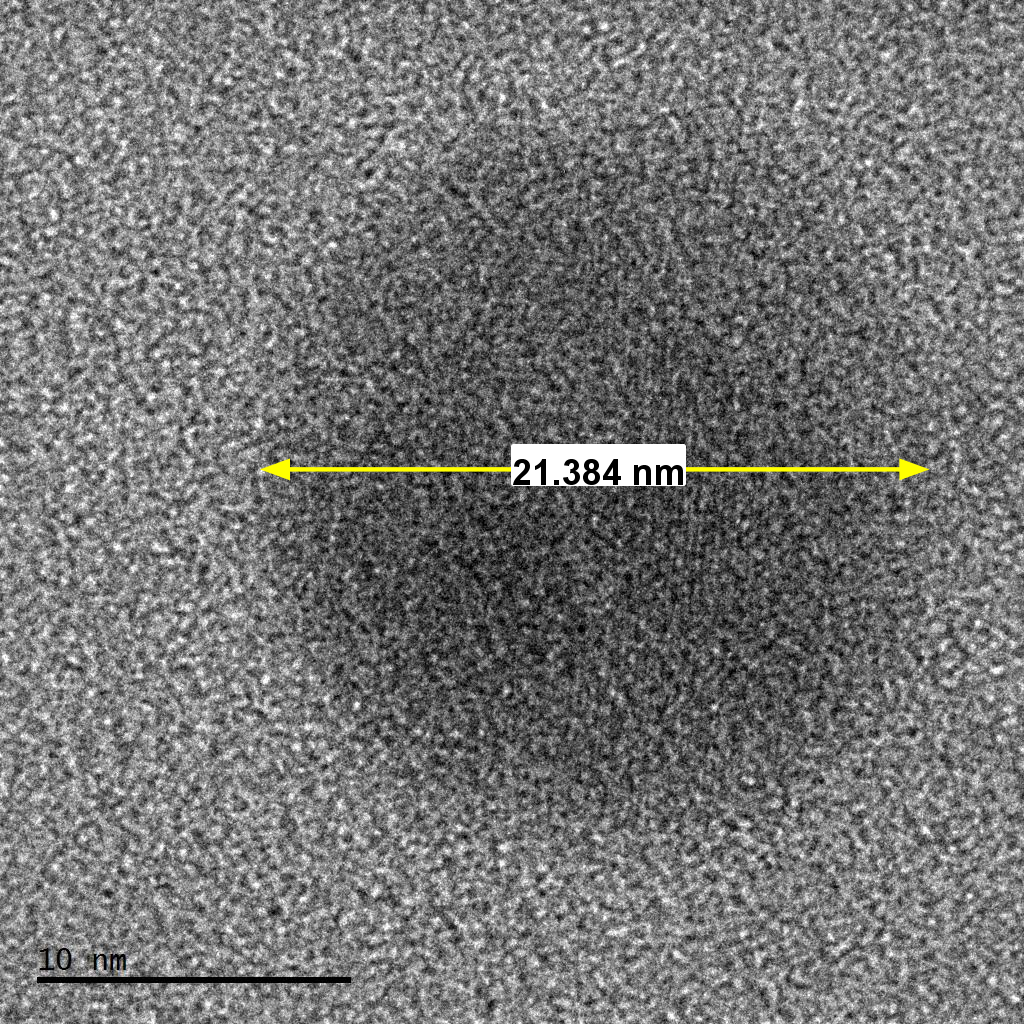


**Fig. S2** TEM images displaying the diameter of CQDs derived from the plant *E. tenuifolia* (Scale bar: 100 nm and 10 nm, respectively).


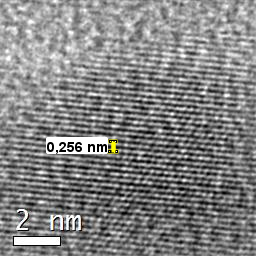


**Fig. S3** The lattice spacing was **calculated** as approximately 0.26 nm **by ImageJ software**, which is close to the lattice spacing of graphite (~ 0.35 nm), indicating the crystalline core of the CQDs from *E. tenuifolia.*

**Table S1:** Phenolic composition (mg/g extract) of the tested extract (nd: not detected).

| **Phenolic components** | **Extract** |
| --- | --- |
| Gallic acid | 2.9 |
| Protocatechuic acid | 0.6 |
| (+)-Catechin | nd |
| *p-*hydroxybenzoic acid | nd |
| Chlorogenic acid | 39.6 |
| Caffeic acid | 2.1 |
| Epicatechin | nd |
| Syringic acid | nd |
| Vanillin | nd |
| *p-*coumaric acid | 1.5 |
| Ferulic acid | 5.2 |
| Sinapic acid | nd |
| Benzoic acid | nd |
| *o-*coumaric acid | nd |
| Rutin | 127.0 |
| Hesperidin | 2.2 |
| Rosmarinic acid | nd |
| Eriodictyol | 1.1 |
| Cinnamic acid | 7.0 |
| Quercetin | 3.3 |
| Luteolin | nd |
| Kaempferol | nd |

**Table S2:** A comprehensive list of the element percentages obtained from XPS analysis.

| ***Name*** | ***Weight %*** |
| --- | --- |
| Si | 6.31 |
| P | 0.59 |
| Cl | 0.48 |
| C | 68.85 |
| N | 0.80 |
| O | 22.96 |


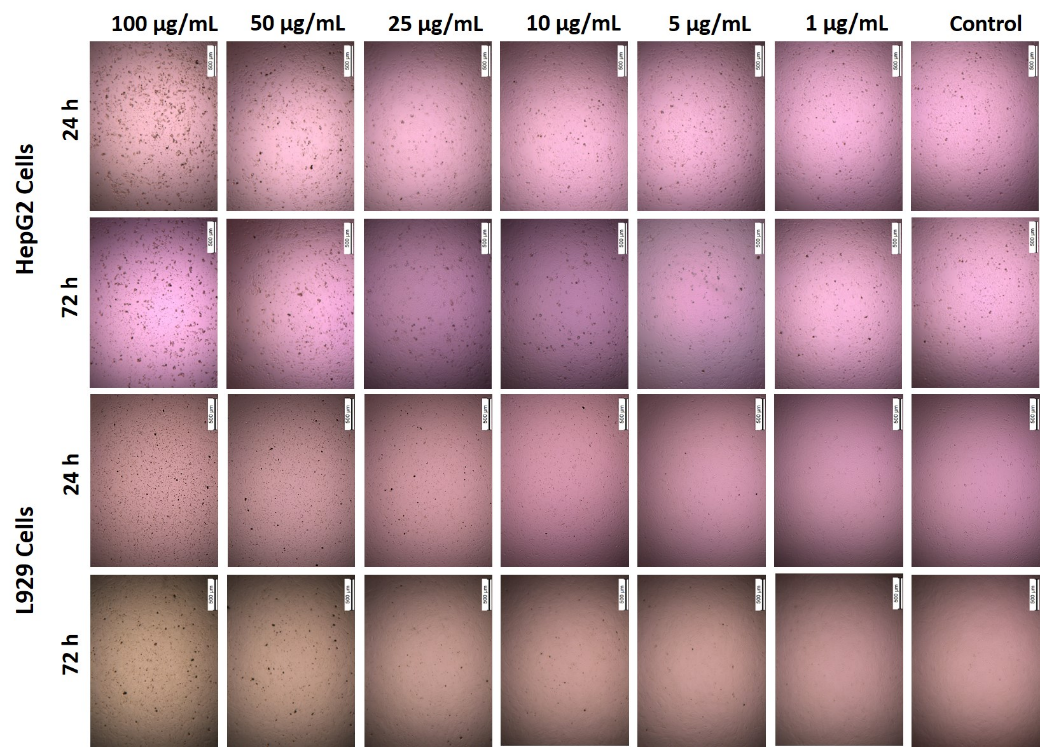


**Fig. S4** Light microscopy imaging of the effects of CQDs on HepG2 and L929 cells.
